# Hitchhiking on Controlled-Release Drug Delivery Systems: Opportunities and Challenges for Cancer Vaccines

**DOI:** 10.3389/fphar.2021.679602

**Published:** 2021-05-10

**Authors:** Lu Han, Ke Peng, Li-Ying Qiu, Meng Li, Jing-Hua Ruan, Li-Li He, Zhi-Xiang Yuan

**Affiliations:** ^1^ College of Pharmacy, Southwest Minzu University, Chengdu, China; ^2^ School of pharmacy, Queen’s University Belfast, Belfast, United Kingdom; ^3^ The First Affiliated Hospital, Guizhou University of Traditional Chinese Medicine, Guiyang, China

**Keywords:** cancer vaccine, *in situ* vaccination, drug delivery system, controlled release, sustained release, hydrogel, microneedle

## Abstract

Cancer vaccines represent among the most promising strategies in the battle against cancers. However, the clinical efficacy of current cancer vaccines is largely limited by the lack of optimized delivery systems to generate strong and persistent antitumor immune responses. Moreover, most cancer vaccines require multiple injections to boost the immune responses, leading to poor patient compliance. Controlled-release drug delivery systems are able to address these issues by presenting drugs in a controlled spatiotemporal manner, which allows co-delivery of multiple drugs, reduction of dosing frequency and avoidance of significant systemic toxicities. In this review, we outline the recent progress in cancer vaccines including subunit vaccines, genetic vaccines, dendritic cell-based vaccines, tumor cell-based vaccines and *in situ* vaccines. Furthermore, we highlight the efforts and challenges of controlled or sustained release drug delivery systems (e.g., microparticles, scaffolds, injectable gels, and microneedles) in ameliorating the safety, effectiveness and operability of cancer vaccines. Finally, we briefly discuss the correlations of vaccine release kinetics and the immune responses to enlighten the rational design of the next-generation platforms for cancer therapy.

## Introduction

Immunotherapy was first introduced by Dr. William Coley to treat malignant tumors using intratumoral injections of live bacteria and bacterial toxins in the 1890s (Coley, 1991). Nowadays, immunotherapy has been fully embraced by the oncologists for the treatment of various tumors. Chimeric antigen receptor (CAR) T cell-based therapies as well as immune checkpoint inhibitors (ICIs), including antibodies targeting programmed cell death protein-1 (PD-1), programmed death-ligand 1 (PD-L1) and cytotoxic T-lymphocyte-associated antigen-4 (CTLA-4), have revolutionized the cancer treatments and have been approved by the U.S. Food and Drug Administration (FDA) for tackling many tumors (Allison, 2015; Sharma and Allison, 2015; Labanieh et al., 2018). However, the response rate to ICIs varies dramatically among cancers. Growing evidence supports the idea that patients lacking pre-existing tumor-infiltrating CD8^+^ T cells have a low response rate to ICIs, suggesting the hypothesis that ICIs need to be combined with other therapies that can stimulate potent tumor-specific T cell responses to improve the clinic outcomes (Hu et al., 2018; van der Burg, 2018; Shae et al., 2019).

One attractive strategy to mount effective antitumor responses is vaccination. Cancer vaccines can provoke antigen-specific immunity or reactivate pre-existing but quiescent tumor-reactive T cells by delivering the antigens and immunoadjuvants with the aim to prevent cancers or fight against established tumor burdens (Lybaert et al., 2018). Two prophylactic vaccines, namely the human papilloma virus (HPV) vaccine and the hepatitis B virus (HBV) vaccine, have been successfully approved for preventing cervical cancer and liver cancer, respectively (Stanley, 2017). Whereas, only one antigen-loaded therapeutic cancer vaccine, termed Sipuleucel-T (Provenge), has received FDA approval for the treatment of advanced prostate cancer so far (Shae et al., 2019). Although cancer vaccines have generated acceptable therapeutic effects in some clinic trials, their overall clinical efficacy is not encouraging especially in solid tumors (Jacobs et al., 2014; Bouzid et al., 2020). The main reasons for these disappointing outcomes include: 1) the use of suboptimal vaccine delivery systems which compromise the vaccine immunogenicity and efficacy; 2) the rapid clearance of antigens at the injection site and the inadequate delivery to lymph nodes; 3) the choice of weakly immunogenic antigens that lack variety and specificity (e.g., overexpressed self-antigens); 4) the inefficient controlling of the immunosuppressive tumor microenvironment (van der Burg, 2018; Shae et al., 2019; Bouzid et al., 2020). On the other hand, cancer vaccines usually require multiple injections to elicit effective immune responses, resulting in poor patient compliance. To surmount these issues, numerous controlled drug release technologies, which are effective in a single shot, have been developed to improve the delivery efficiency and potency of cancer vaccines. Controlled-release platforms can prolong the spatiotemporal presentation of antigens and immunomodulators to immune cells or mimic the prime-boost effect of the traditional multi-bolus vaccination schedules, thereby stimulating stronger antitumor immune responses (Ali et al., 2009b; Korupalli et al., 2019; Irvine et al., 2020). Moreover, the controlled-release systems can also improve patient compliance by eliminating the booster shots, minimize the toxic side effects by reducing drug dose and avoiding rapid drug clearance, as well as improve outcomes by integrating the synergistic effect of multiple therapeutics within one platform (Huang et al., 2019; Riley et al., 2019). Especially for *in situ* cancer vaccines that require intratumoral injections, this single-dose vaccine technology can improve the antitumor immunity and increase the operational feasibility by a local and sustained release of immunomodulators *in vivo*.

Below, we will present recent advances in the engineering of controlled-release delivery platforms for improving the safety and efficacy of cancer vaccines. Firstly, we introduce the advantages and challenges of various types of cancer vaccines. Secondly, an overview of the controlled-release drug delivery systems is provided and the limitations that should be addressed to improve the safety, effectiveness and operability of cancer vaccines are enlightened. Finally, we briefly discuss the possible relevancy between the release kinetics of controlled-release vaccines and the type and magnitude of the immune responses.

## Conventional Cancer Vaccines

Conventional vaccines are composed of antigens and adjuvants. The selection of an immunogenic antigen plays a critical role in implementing the vaccine effectiveness and specificity. Tumor antigens can be broadly divided into two categories: tumor-associated antigens (TAAs) and neoantigens. TAAs are overexpressed self-antigens that are shared among many tumors and can also be found in some normal cells. TAAs have been widely used in cancer vaccines for decades but they are not tumor-specific antigens and can only trigger weak immune responses owing to thymic tolerance (Hu et al., 2018). In contrast, neoantigens are highly tumor-specific immunogens derived from somatic mutations and they are more immunogenic due to the lack of central tolerance (Chu et al., 2018). With the rapid development of next-generation sequencing and peptide immunogenicity prediction technologies, neoantigens represent the most promising candidates for the preparation of personalized cancer vaccines and the diagnosis of ICI therapy response (Desrichard et al., 2016). Tumor antigens can be presented in various forms ranging from defined proteins, peptides, protein-encoding DNA or RNA, recombinant viral or bacterial vectors, tumor cell-based preparations, to antigen-pulsed dendritic cells (DCs).

The other crucial component of a vaccine is the adjuvant that can strengthen the magnitude of the immune responses or skew the immune responses toward Th1 or Th2 immunity. There are several types of vaccine adjuvants, including toll-like receptor (TLR) agonists, e.g. unmethylated cytosine-phosphate-guanine (CpG), imiquimod and polyinosinic:polycytidylic acid (poly(I:C)); cytokines, e.g. interferon (IFN)-α and interleukin (IL)-12; bacterial derivatives, e.g. monophosphoryl lipid A (MPLA) and Bacille Calmette-Guerin (BCG); depot-like adjuvants, e.g. aluminum salts, incomplete Freund’s adjuvant (IFA) and Montanide; particle adjuvants, e.g., liposomes, virus-like particles and polymeric particles (Sivakumar et al., 2011; Pilla et al., 2018). However, only aluminum salts, three emulsions (MF59, AS03, AF03), virosomes and MPLA are approved for human vaccines (Lybaert et al., 2018). Therefore, exploring potent and safe adjuvants and identifying the optimal adjuvants for vaccines are also appealing areas of research.

### Subunit Vaccines

Subunit vaccines are defined by the use of purified proteins, peptides or polysaccharides as the antigens to stimulate the immune responses. Subunit vaccines usually contain one or several well-structured antigenic components, making them easier to manufacture and much safer than live pathogen vaccines, as well as avoiding the side effects from antigen-induced unrelated immune responses (Table 1) (Riley et al., 2019). However, these vaccines can hardly conquer the tumor heterogeneity between and within tumors as a consequence of lacking antigen variety. One strategy is mixing multiple antigens in one formulation to benefit the antitumor immune responses (Fennemann et al., 2019; Noguchi et al., 2020). In comparison of whole protein-based vaccines, peptide vaccines only contain one or several epitopes, which can be easily processed by DCs (Rosalia et al., 2013). Peptide vaccines are composed of TAA peptides or neoantigen peptides which are presented in the form of short peptides (<15 amino acids) or long peptides (15–40 amino acids), and the difference between the short peptides and the long peptides has been elucidated in detail elsewhere (Lybaert et al., 2018; Bouzid et al., 2020).

**TABLE 1:** Characteristics of conventional cancer vaccines and *in situ* cancer vaccines.

Therapy	Classification	Advantages	Disadvantages
Subunit vaccines	Protein vaccines	Easy for mass production	Weak immunogenicity
	Peptide vaccines	Cheap	Lacking antigen variety
		Safe to use	Short peptides: HLA-restricted
		Long peptides: not HLA-restricted	
		Neoantigen peptides can be personalized	
Genetic vaccines	DNA vaccines	Easy for mass production	Weak immunogenicity
	RNA vaccines	Cheap	Rapid degradation
		Can encode multiple antigens	Limited cellular transfection
		Not HLA-restricted	DNA vaccines have the risk of integration into the host genome
Tumor cell-based vaccines	Autologous tumor cell vaccines	Contain the whole tumor antigens	Complex preparation process
	Allogeneic tumor cell vaccines	Autologous vaccines: not HLA-restricted	Weak immunogenicity
		Allogeneic vaccines have a broader target population	May have immunosuppressive effects
			May induce autoimmunity
			Autologous vaccines need tumor biopsies or operation
DC vaccines	Exogenous DC vaccines	Exogenous DCs: safe; measurable maturation	Exogenous DCs: costly; complex preparation process; short shelf-life
	Endogenous DC-targeting vaccines	Endogenous DC vaccines: easy to fabricate; can program a large scale of DC subsets	Not fully activated DCs may induce immune tolerance
	Artificial DC vaccines	Artificial DCs: long shelf-life; not vulnerable to the tumor immunosuppressive conditions	
In situ cancer vaccines	—	Simple, personalized and off-the-shelf	Need intratumoral injections
		No need for identification and isolation of tumor antigens	Weak immunogenicity (need combination therapies)
		Contain the whole tumor antigens	

Despite these excellent features, the major challenge that hinders the broad application of subunit vaccines is the low immune efficacy, which is partially due to the poor uptake of antigens and adjuvants in lymph nodes (Pilla et al., 2018; Riley et al., 2019). To overcome this issue, vaccines need to be exquisitely engineered using the following strategies. 1) Increasing the accumulation of antigens and adjuvants in lymph nodes. This can be achieved by a direct intra-lymph node injection (Ribas et al., 2011), especially when immunogenic cargos are loaded in sustained-release platforms that allow extended retention in lymph nodes (Shae et al., 2019). Another approach is the use of albumin-hitchhiking vaccines which can be constructed through conjugating an antigen (or an adjuvant) to a lipophilic albumin-binding tail (Liu et al., 2014) or to a derivative of Evans Blue (Zhu et al., 2017). Besides, nanoparticles with optimized size, surface charge and composition are more likely to be drained into lymph nodes (Jiang et al., 2017). 2) Promoting antigen uptake by DCs, such as using anchoring endocytosis molecules (e.g., mannose, fucose and N-acteylglucosamine)-decorated nanoparticles (Koerner et al., 2019) or antibody-antigen conjugates (Keler et al., 2007). 3) Improving the adjuvant effect of vaccines. This can be implemented by adding more powerful adjuvant reagents or using delivery vehicles with adjuvant activity, such as liposomes or nanoparticles (Vartak and Sucheck, 2016). 4) Promoting cross-presentation of the antigens to potentiate the Th1 immune responses. For example, pH-responsive cationic polymers can facilitate endosomal antigen escape by the “proton sponge” effect (Shae et al., 2019).

### Genetic Vaccines

Genetic vaccines have emerged as promising alternatives to subunit vaccines by exploiting tumor antigen-encoding DNA or RNA sequences that require intracellular delivery into DCs to express targeted antigens (Pardi et al., 2018). Genetic vaccines have many merits (Table 1), such as cost-effectiveness, easy for mass production, allowing the delivery of multiple antigens in one platform, and avoiding human leukocyte antigen (HLA) restriction (Lybaert et al., 2018). In spite of these promising features, the clinical results of plasmid DNA vaccines for treating solid tumors have been disappointing mainly due to the barriers for nuclear delivery, low immunogenicity and the immunosuppressive factors within the tumor (Lopes et al., 2019; Riley et al., 2019). Alternatively, mRNA-based vaccines only require to cross the cell membranes to be translated in the cytoplasm, which avoids the risk of integration into the host genome (McNamara et al., 2015). Moreover, the *in vivo* half-life and immunogenicity of mRNA can be regulated by modification and delivery strategies (Pardi et al., 2018). However, the rapid degradation of mRNA by nucleases and the limited translocation into the cytoplasm still challenge the development of RNA-based vaccines (Lybaert et al., 2018; Lin et al., 2020).

Based on the characteristics of genetic vaccines, delivery systems with the ability to increase the drug delivery into lymph nodes, promote intracellular uptake of nucleic acids, facilitate protein translation and reduce nucleic acid degradation will greatly prompt the clinical application of genetic vaccines. These vaccines can be delivered in diverse ways, for instance, by gene gun, electroporation, ultrasound, laser, viral or bacterial vectors, liposomes, nanoparticles, autologous DCs or other carrier modalities (Guo et al., 2013). The safety and efficacy of various viral vectors, such as pox and adenovirus, have been tested in clinical trials, but the high immunogenicity of viral vectors can, on the contrary, lead to the secretion of neutralizing antibodies (Pilla et al., 2018). A heterologous prime-boost regimen can handle this dilemma. For example, PROSTVAC was a promising viral cancer vaccine, composed of a vaccinia priming vector and a fowlpox boosting vector carrying transgenes for human prostate-specific antigen (PSA) along with three costimulatory molecules (CD80, CD58, and CD54), but it failed to benefit the patients in a phase III study (Gulley et al., 2019; Zhao et al., 2019). Cationic materials are commonly used to condense nucleic acids. For example, a lipid nanoparticle with ionizable lipids was allowed to form complexation with negatively charged mRNA encoding two TAAs (gp100 and TRP2) at low pH, and this formulation could induce a strong CD8^+^ T cell response and remarkable tumor shrinkage (Oberli et al., 2017). Moreover, a local gene depot made of mRNA polyplex-loaded implantable porous scaffolds showed superior sustained delivery and higher local transgene expression than a bolus injection (Chen et al., 2018). This indicates that sustained delivery of mRNA may potentiate the antitumor immunity. Furthermore, the combination of immunoadjuvants or other cancer therapies may further boost the therapeutic effects of genetic vaccines.

### Tumor Cell-Based Vaccines

In addition to neoantigens, another class of patient-individualized antigens is whole tumor cell derivatives including the live attenuated tumor cells, killed tumor cells, tumor lysates, tumor-derived exosomes, tumor-derived whole RNAs, tumor cell membrane-based particles, and fusions of tumor cells and DCs (Browning, 2013; Fang et al., 2014; Chiang et al., 2015). Autologous tumor cell vaccines based on patient individual-derived cancer tissues, contain the complete antigen repertoire of the tumor, thereby avoiding the costly and complex identification procedure needed for neoantigens (Table 1). Even more importantly, they are not restricted to HLA type. However, the preparation of whole tumor cell vaccines requires multiple steps: 1) obtaining the patient’s tumor cells by surgery or biopsy, 2) processing them *in vitro* to acquire tumor cell derivatives, 3) then loading the tumor antigens into delivery systems. These complex procedures may hinder the clinical practicality of tumor cell-based vaccines. Moreover, this autologous vaccine technology is only feasible for the patients with selected tumor types and stages that are capable of tumor biopsies or operation (Schlom et al., 2014). Alternatively, allogeneic tumor cell vaccines can benefit more patients with homologous tumors because they are prepared from two or three specific tumor cell lines which have been established and characterized (Schlom et al., 2014). In one example of this, Canvaxin containing three irradiated melanoma cell lines showed excellent therapeutic results in phase II clinical trials, but it did not meet the expectation in phase III trials (Ozao-Choy et al., 2014). This probably resulted from the use of suboptimal dosage, schedule and adjuvant. Tumor stroma-associated antigens are another kind of attractive targets for cancer vaccines as they are genetically more stable and less subjected to tumor immune evasion mechanisms in contrast to tumor cells (Chiang et al., 2015).

Although tumor cell-based vaccines have attracted enormous attention in recent years, two of the most important challenges for whole tumor cell vaccines remain the low accumulation in lymph nodes and low immunogenicity. Numerous strategies have been developed to handle these issues, such as cloaking nanoparticles with tumor cell membranes or fused cytomembranes derived from tumor cells and DCs (Liu et al., 2019b; Gan et al., 2020), or loading the immunogenically dying tumor cells with adjuvants (e.g., BCG) or adjuvant-loaded nanoparticles (Fan et al., 2017). For example, the sustained release of adjuvants and tumor cell membrane-coated nanoparticles from thermosensitive hydrogels could recruit DCs and induce a strong CD8^+^ T cell response, and the combination with antibodies targeting PD-1 could further prolong the survival time of tumor-bearing mice (Ye et al., 2019). It is worth noting that the immune properties of tumor-derived exosomes can be immune-activating or immunosuppressive depending on the physiological state of donor cells and exogenous factors (Théry et al., 2009; Greening et al., 2015). Fortunately, the immunosuppressive role of tumor-derived exosomes can be switched to promote the antitumor immune responses by using heat treatment, adding strong adjuvant components or using transgenic tumor cells expressing IL-2 or IL-18 (Cho et al., 2009; Théry et al., 2009). Besides, the whole tumor cell-based vaccines also contain abundant normal self-proteins which may induce autoimmunity as well as diminish the vaccine effectiveness by the dilution of the most immunogenic tumor antigens (Obeid et al., 2015; Hu et al., 2018). Using autologous induced pluripotent stem cells as the antigen resource may provide new insights to tackle these obstacles (Ouyang et al., 2019).

### DC Vaccines

DCs, as the most potent professional antigen-presenting cells (APCs), play a pivotal role in initiating and bridging the innate and adaptive immune responses. The ultimate goal of many delivery strategies of cancer vaccines is to target DCs and fully activate them. One approach for this is exogenous DC vaccines which usually rely on the isolation and differentiation of peripheral blood autologous DC precursor cells into immature DCs, followed by loading these immature DCs with a proper form of antigens (such as peptides, proteins, nucleic acids or tumor cell derivatives), then maturating these DCs with different stimuli, and finally reinfusing the manipulated DCs to the patient to implement the cancer immunotherapy (Bol et al., 2016; Sabado et al., 2017). Sipuleucel-T, prepared from autologous DCs loaded with a fusion protein (PA2024) of prostatic acid phosphatase (PAP) and granulocyte-macrophage colony-stimulating factor (GM-CSF), was approved in 2010 to treat metastatic prostate cancer on the basis of a survival benefit (Gardner et al., 2012). Although substantial clinical studies have demonstrated the safety and immunogenicity of exogenous DC vaccines, the clinical outcomes are not encouraging (Perez and De Palma, 2019). Many factors affect the efficacy of exogenous DC vaccines, including a suboptimal choice of DC subsets, limited migration to the lymph nodes and negative immune regulation within tumors. Hence, DC vaccines can be further improved by optimizing, for instance, the choice of DC type and antigen type, activation method, the design of drug delivery system, the number of DCs for injection, the vaccination schedule and the administration route (Sabado et al., 2017). Another approach to exploit natural DCs in cancer vaccines is targeting and modulating the endogenous DC subpopulations *in vivo.* This targeting goal can be implemented by various strategies involving GM-CSF-secreting irradiated tumor cells, conjugation of antigens or antigen-loaded nanoparticles to ligands directing against DC surface receptors (e.g., CD40, DEC205, Langerin or Clec9A), or using injectable controlled-release platforms that are engineered to release chemokines, tumor antigens and adjuvants (Sabado et al., 2017; Calmeiro et al., 2019). These *in vivo* targeting technologies allow to program a large scale of DC subsets without the need for a costly and labor-intensive extracorporeal training of exogenous DCs (Table 1; Sabado et al., 2017). However, the endogenous DC-targeting vaccines cannot control the extent of antigen loading and DC activation as the exogenous DC vaccines do, and DCs that are not fully activated may induce immune tolerance (Bouzid et al., 2020). Recently, artificial DCs, such as nanoparticles coated with tumor lysate-primed DC membranes or scaffolds carrying T cell activation cues and peptide antigens, can directly stimulate the activation and expansion of antigen-specific T cells (Cheung et al., 2018; Cheng et al., 2020). These artificial DCs can circumvent the short shelf-life concerns of exogenous DC vaccines and they are not vulnerable to the tumor immunosuppressive conditions (Cheng et al., 2020). Furthermore, DC vaccine-based combination therapies may provide better control over cancers (Bol et al., 2016; Tanyi et al., 2018), thus many clinical trials are exploring the synergistic effect of DC vaccines and ICI therapies (Sprooten et al., 2019).

## *In Situ* Cancer Vaccines

**FIGURE 1:**
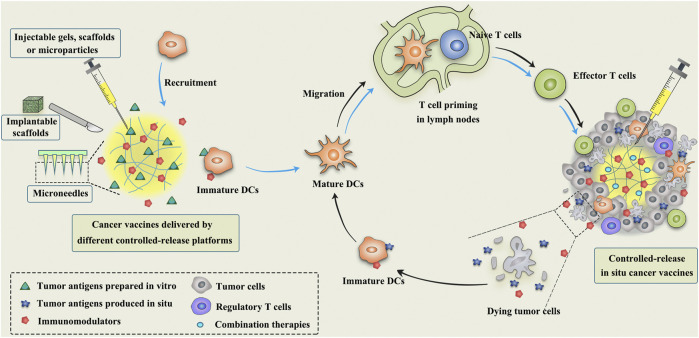
Schematic illustration of the mechanism of controlled-release cancer vaccines for cancer therapy. The blue arrows indicate the antitumor process of conventional cancer vaccines, while the black arrows indicate the mechanism of *in situ* cancer vaccines.

Despite the fact that the substantial improvement achieved by conventional cancer vaccines, the risk of undetected contaminations during the elaborate preparation process and the high costs associated with preparation and storage remain bottlenecks limiting their broad clinical implementation (Duong et al., 2020a). *In situ* vaccination (ISV), without the need for previous identification and isolation of tumor antigens, is raising great attention in cancer immunotherapy. ISV is a simple, personalized and off-the-shelf cancer vaccine by *in vivo* transforming tumors into “antigen factories” (Table 1). As depicted in Figure 1, ISV can activate systemic antitumor immune responses by simply delivering immunomodulators (Hammerich et al., 2015; Sheen and Fiering, 2019). Taking advantage of the complete antigenic repertoire of a tumor, including all mutated antigens and unmutated antigens, ISV potentiates the immune system to recognize the evolving tumor antigen arrays, thereby overcoming the weak immunogenicity of single-antigen vaccines and minimizing immune escape.

Broadly speaking, any approach that employs TAAs available at the tumor site to elicit antitumor immune responses can be termed as ISV (Hammerich et al., 2015). ISV usually refers to the approaches using intratumoral administration of immunomodulators to activate immune cells or reverse the immunosuppressive microenvironment within the tumor. The most extensively studied immunomodulators include bacteria and its derivatives (Janku et al., 2021), oncolytic viruses (Oh et al., 2017; Russell and Barber, 2018), TLR agonists (Wei et al., 2019), monoclonal antibodies (e.g., anti-PD-1, anti-CTLA-4 and anti-CD40) (Rahimian et al., 2015; Wang et al., 2016; Knorr et al., 2018), immunomodulatory cytokines (e.g., IL-2 and IL-12) (Jackaman and Nelson, 2012; Hwang et al., 2020), and immune cells (e.g., DCs) (Subbiah et al., 2018). In an example of successful ISVs, Talimogene laherparepvec (T-VEC), a genetically modified herpes simplex virus that can replicate selectively within tumors and express GM-CSF, is the first intratumoral oncolytic viral therapy approved by the FDA in 2015 for local treatment of unresectable advanced melanoma (Rehman et al., 2016).

However, a single regimen of immunomodulators is generally difficult to surmount the complexity and compensatory evolution of tumors, especially since many types of tumor cell death are nonimmunogenic, thus leading to suboptimal clinic outcomes (Sheen and Fiering, 2019). Local treatment with radiotherapy, phototherapy, oncolytic viruses, cryoablation, sonodynamic therapy, or some chemotherapeutic drugs (e.g., anthracyclines, taxane, cyclophosphamide, gemcitabine and oxaliplatin) can induce significant immunogenic cell death (ICD) of tumor cells when administrated at appropriate doses and schemes (Pol et al., 2015; Galluzzi et al., 2017; Min et al., 2017; Twumasi-Boateng et al., 2018; Yakkala et al., 2019; Zhao et al., 2020; Zhu et al., 2020). ICD can expose calreticulin on the surface of dying tumor cells to provide an “eat-me” signal for APCs, and release a large number of damage-associated molecular patterns (DAMPs) to provide adjuvant stimuli to activate APCs (Galluzzi et al., 2017). Therefore, the combination of ICD-inducing therapies with immunomodulators, such as chemoimmunotherapy, radioimmunotherapy and photoimmunotherapy, can not only directly kill tumor cells, but also turn ‘cold’ tumors into ‘hot’ tumors to elicit potent immune responses against a broad spectrum of cancers. In an elegant example of this, Patel et al. combined bacterial membrane-coated nanoparticles (BNP) with radiotherapy to treat syngeneic melanoma or neuroblastoma in mice (Patel et al., 2019). This multifunctional BNP consisted of an immunostimulatory PC7A/CpG polyplex nanocore coated with bacterial membrane and imide groups. After radiotherapy, BNP could capture TAAs released by ICD, followed by promoting the antigen uptake and cross presentation. Subsequently, a strong antitumor T-cell response was induced, leading to remarkable tumor regression alongside immunological memory.

Although ISV and its combinatorial therapies are gaining rising attention among researchers, the magnitude and durability of antitumor immune responses induced by ISV could be unfortunately impeded by the immunosuppressive tumor microenvironment (van der Burg et al., 2016). One appealing strategy is to introduce CAR-T, ICIs or other immunosuppressive signal inhibitors in the combinatorial therapies to improve the outcomes (Wang et al., 2018; Chen et al., 2019a; Song et al., 2019; Chen et al., 2020a). Moreover, approaches that loosen the desmoplastic stroma or target the tumor vasculature would help to augment the antitumor immunity of ISV by facilitating immune infiltration (Galluzzi et al., 2018). Furthermore, identifying the optimal immunoadjuvants and applying the most suitable delivery system may maximize the safety and effectiveness of ISV. Although intratumoral injection technology is widely used in accessible superficial tumors, such as skin, head and neck, and breast tumors, it remains a challenge for those deep tumors, such as brain, liver and pancreas tumors (Sheen and Fiering, 2019). Fortunately, with the help of modern imaging technologies such as ultrasound, computed tomography guidance and laparoscopy, safe and accurate injections can be performed for tumors in various locations (Sheen and Fiering, 2019). Moreover, one should take full account of the effects of injection pressure, volume, viscosity, frequency, etc. on tumor bulk, therapeutic effect and patient tolerance. Collectively, combinatorial ISV therapies have great potential to provide a comprehensive approach to treat heterogeneous cancers.

## Controlled-Release Drug Delivery Systems

Early depot-like adjuvants, such as aluminum salts and IFA, are effective in promoting protective humoral immunity against pathogens, but they are not excellent candidates for cancer vaccines that rely on cellular immunity (Brito and O'Hagan, 2014; Shae et al., 2019). Therefore, the next generation of controlled-release cancer vaccines should be able to elicit efficient and durable antitumor cellular immune responses. To date, numerous controlled-release delivery platforms, such as polymeric microspheres, scaffolds, hydrogels and microneedles (MNs) have been designed for local and controlled release of multiple immunotherapeutic agents to improve antitumor efficacy and reduce off-target toxicities (Figure 1; Table 2).

**TABLE 2:** Representative examples of cancer vaccines delivered by controlled-release platforms (from 2017 to 2021).

Delivery system	Composition	Antigens	Adjuvants and combination therapies	Tumor model	Reference
1. Subunit vaccines					
MPs	Mesoporous silicon vector	TRP2 peptide	CpG and MPLA	C57BL/6 mice with B16 tumor	Zhu et al. (2018)
Injectable Scaffolds	PEI-coated Mesoporous silica rods	OVA or neoantigen peptides	GM-CSF, CpG and anti-CTLA4	C57BL/6 mice with E7-TC-1, B16F10, or CT26 tumor	Li et al. (2018)
Injectable hydrogels	***Comp.1*** peptides	OVA	—	C57BL/6 mice with EG7-OVA or B16-OVA tumor	Wang et al. (2020b)
MNs	Pluronic F127	OVA	Resiquimod	C57BL/6 mice with EG7-OVA tumor	Kim et al. (2018)
2. Genetic vaccines					
Scaffolds	Mesoporous silica microrods	DNA polyplexes encoding OVA	GM-CSF, PEI, CpG and anti-PD-1	C57BL/6 mice with B16-OVA tumor	Nguyen et al. (2020)
Injectable hydrogels	HA-PCLA	DNA polyplexes encoding OVA	GM-CSF and PEI	C57BL/6 mice with B16-OVA tumor	Duong et al. (2020a)
MNs	PVA	RALA/pDNA nanoparticles encoding PSCA	—	C57BL/6 mice with TRAMP-C1 tumor	Cole et al. (2019)
3. Tumor cell-based vaccines					
MPs	Yeast derived β-glucan	Tumor cell lysate	CpG	C57BL/6 mice with MC38 tumor	Hou et al. (2021)
Scaffolds	Collagen and HA	Tumor cell lysate	Nanogel-based poly (I:C) and gemcitabine	BALB/c mice with 4T1 tumor	Phuengkham et al. (2018)
Injectable hydrogels	Tumor-penetrable peptides	Dead tumor cells	ICG and JQ1	BALB/c mice with 4T1 tumor or EMT6 tumor	Wang et al. (2018)
Injectable hydrogels	HA and Pluronic F-127	Tumor cell membrane-coated BPQD nanovesicles	GM-CSF, LPS, anti-PD-1 and NIR irradiation	BALB/c mice with 4T1 tumor and C57BL/6 mice with B16F10 tumor	Ye et al. (2019)
MNs	HA	Tumor cell lysate	GM-CSF, melanin and NIR irradiation	C57BL/6J mice with B16F10 or BPD6 tumor; BALB/cJ mice with 4T1 tumor	Ye et al. (2017)
4. DC-based vaccines					
Injectable hydrogel	RADA16 peptide	OVA-pulsed DCs + free OVA or tumor cell lysate-pulsed DCs + tumor cell lysate	anti-PD-1	C57BL/6 mice with EG7-OVA tumor	Yang et al. (2018)
Scaffold	Mesoporous silica micro-rods	APC-mimetic scaffold presenting tumor peptides, CD28 and IL-2	19BBz CAR-T cells	NSG mice with Raji xenograft tumor	Cheung et al. (2018)
5. *In situ* cancer vaccines					
MPs	Polylactic acid	—	IL-12 and stereotactic body radiation	C57BL/6J and KPC mice with KOKC or *Pan*02 tumor	Mills et al. (2019)
Injectable hydrogels	Alginate	—	GM-CSF, CpG and doxorubicin-iRGD conjugate	BALB/c mice with 4T1 tumor	Wang et al. (2020a)
Injectable hydrogels	Gelatin-hydroxyphenyl propionic acid	—	Exogenous DCs and oncolytic adenovirus co-expressing IL-12 and GM-CSF	C57BL/6 mice with LLC tumor	Oh et al. (2017)
MNs	PVA and PVP	—	1-methyl-tryptophan, chitosan nanoparticles containing ICG and NIR irradiation	C57BL/6 mice with B16 tumor	Chen et al. (2020a)

—, not performed; MPs, microparticles; TRP2, tyrosinase related protein 2; CpG, unmethylated cytosine-phosphate-guanine; MPLA, monophosphoryl lipid A; OVA, ovalbumin; GM-CSF, granulocyte-macrophage colony-stimulating factor; PEI, polyethyleneimine; anti-CTLA-4, cytotoxic T-lymphocyte-associated antigen-4 antibody; MNs, microneedles; PLGA, poly(lactide-co-glycolide); IL, interleukin; DNA, deoxyribonucleic acid; anti-PD-1, programmed cell death protein-1 antibody; HA-PCLA, levodopa- and poly(ε-caprolactone-co-lactide)ester-functionalized hyaluronic acid; poly(I:C), polyinosinic:polycytidylic acid; PVA, poly(vinyl pyrrolidone); pDNA, plasmid DNA; RALA, cationic peptide consists of arginine/alanine/leucine/alanine repeats; HA, hyaluronic acid; APC, antigen-presenting cell; PSCA, prostate stem cell antigen; TRAMP-C1, transgenic adenocarcinoma mouse prostate cell line 1; LPS, lipopolysaccharides; anti-PD-L1, programmed death-ligand 1 antibody; BPQD, black phosphorus quantum dot; NIR, near-infrared; DC, dendritic cell; CAR-T cells, chimeric antigen receptor T cells; iRGD, an internalizing cyclic peptide containing an Arg-Gly-Asp (RGD) motif; PVP, poly(vinyl alcohol); ICG, indocyanine green.

### Microparticles

**FIGURE 2:**
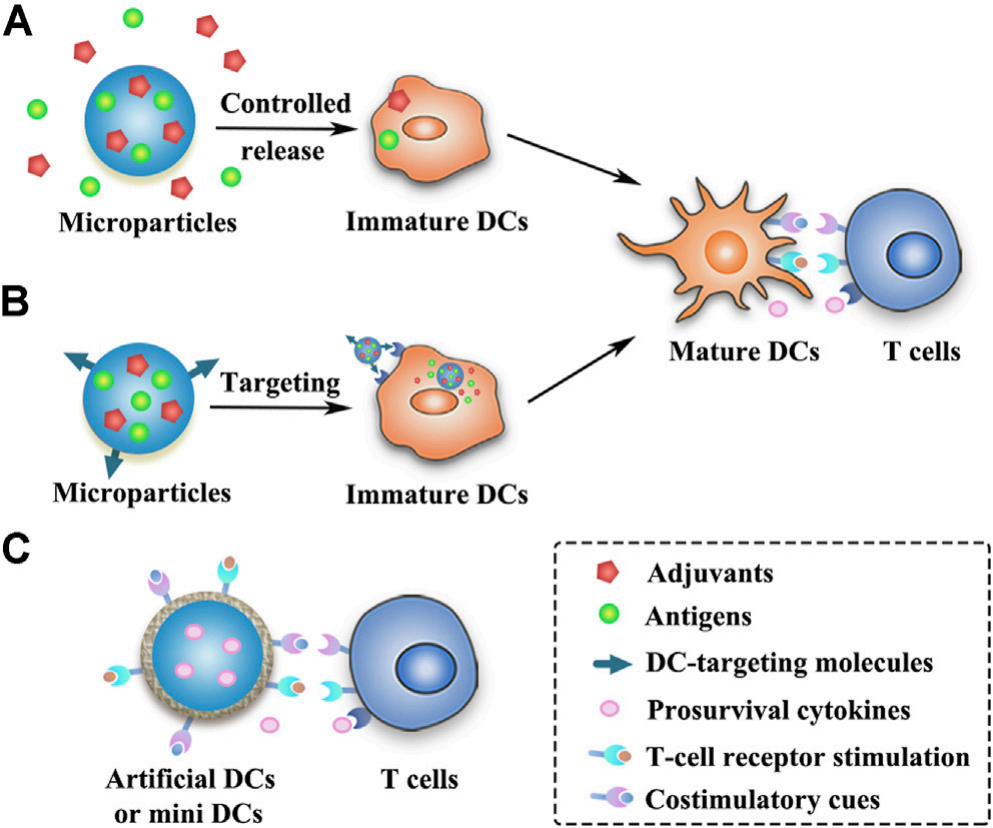
Schematic presentation of strategies of different MP-based cancer vaccines. **(A)** Antigens and adjuvants are released in a controlled manner from the MPs and then taken up by the immature DCs. **(B)** DC-targeting MPs can release antigens and adjuvants within the DCs after being taken up by the immature DCs. **(C)** MP-based artificial DCs or mini DCs can present T-cell activation signals to activate T cells or directly expand primary T cells.

Peris and Langer proposed in 1979 for the first time that the release of antigens could be controlled using polymeric materials to stimulate immune responses (Peris and Langer, 1979). Numerous natural and synthetic biodegradable polymeric materials including chitosan, alginate, gelatin, poly(lactic-co-glycolic acid) (PLGA), Poly(lactic acid) (PLA), poly(ε-caprolactone) (PCL), poly(β-amino esters) (PBAE) and poly(methyl methacrylate) (PMMA), etc., as well as some inorganic materials (such as silica) have been widely used in the field of controlled release of antigens and immunomodulatory agents (Lin et al., 2015; Zhu et al., 2018; Huang et al., 2019; Koerner et al., 2019; Hou et al., 2021). Polymeric microparticles (MPs) or microspheres can induce potent antigen-specific immunity by controlling the release of antigens or mimicking the size of pathogens, but they are much safer than live pathogens (Huang et al., 2019). Different forms of antigens as mentioned above (e.g., purified proteins, peptides, nucleic acids, and cell lysates) have been successfully formulated in MPs (Joshi et al., 2014; Pradhan et al., 2014; Guarecuco et al., 2018; Zhu et al., 2018) (Table 2). As compared to bolus injections, MPs can protect the antigens and adjuvants from degradation with slow release at the injection site to prolong antigen presentation and allow simultaneous delivery of antigens and adjuvants to the same APC (Lin et al., 2015; Huang et al., 2019) (Figure 2). By modulating the polymer molecular weight, composition, preparation method, particle size and additives, etc., MPs can provide sustained or pulsatile release of entrapped antigens over periods lasting weeks to months (Sivakumar et al., 2011). Furthermore, the modification of surface physicochemical properties or decoration with ligands or antibodies can lead to different functionalized MPs (Figure 2), such as APCs-targeting MPs, immune cell-engaging particles (artificial DCs) or MPs for intranasal vaccination (Mata et al., 2011; Li et al., 2016b; Jung et al., 2019; Koerner et al., 2019; Huang et al., 2020).

Among the most extensively exploited synthetic polymers in many areas, PLGA has been approved by the FDA for human use in drug delivery and biomedical devices due to its biodegradability and biocompatibility (Koerner et al., 2019). PLGA micro- or nanoparticles are promising carriers with remarkable application prospects for cancer vaccines. For example, tumor lysates alongside with TLR agonists (CpG-ODN) were co-encapsulated into PLGA microspheres to induce cytotoxic T cell responses and mediate tumor shrinkage in a transgenic mouse model of prostate cancer, and these particles could be sterilized by *γ*-irradiation without impairing their antitumor efficacy (Mueller et al., 2012). Recently, PLGA microspheres loaded with antigens and photosensitizers facilitated an active transport of microsphere-containing APCs to draining lymph nodes after illumination, resulting in strong CD8^+^ T cell responses (Schineis et al., 2021). Moreover, combination therapies that attack tumors from all sides can further augment the antitumor effects of MPs-based vaccines. PLGA nanoparticles that co-deliver doxorubicin, two immune adjuvants (poly(I:C) and R848), and one chemokine (CCL20) generated superior antitumor effects than separate compounds, as demonstrated on two treatment-resistant lung and colon cancer models (Silva et al., 2019). However, the prolonged survival time requires four repeated intratumoral injections of the PLGA nanoparticles, indicating that MPs may be a preferable platform for the sustained delivery of multiple drugs.

In spite of these promising features of PLGA MPs, the encapsulation efficiency and loading efficiency of hydrophilic drugs in PLGA MPs are usually low, and antigens, especially the macromolecular proteins, have high risks of aggregation or degradation in the presence of organic solvents and high shear stresses during the preparation process, leading to impaired antigenicity and immunogenicity. Many strategies have been developed to maintain the integrity of proteins. Bailey et al. developed a “self-healing encapsulating” method to load protein antigens into pre-made porous PLGA microspheres with minimal impact on the antigens (Bailey et al., 2017). Besides, before encapsulated into the PLGA MPs, protein antigens can be previously loaded into polysaccharide (dextran) glassy particles through freezing-induced phase separation to protect antigen’s integrity (Geng et al., 2008). In another example, antigens and other immunomodulators can be efficiently and intactly loaded on the surface of PLGA MPs by conjugating short synthetic DNA scaffolds to the surface (Huang et al., 2020). More importantly, many advanced manufacturing processes, such as spray drying technology and supercritical carbon dioxide, are able to greatly improve the drug loading, protein stability, reproducibility and scaling-up production of PLGA MPs (Han et al., 2016a; Koerner et al., 2019). Another challenge limiting the broad application of PLGA MPs as vaccine carriers is high initial burst release that often consumes about 25% of the total drug in the first day (Park et al., 2019). This initial burst release, mainly resulted from the quick dissolution of adsorbed or weakly bound drugs on the surface of PLGA MPs, may lead to unintentional toxicity (Koerner et al., 2019). The release kinetics of PLGA MPs can be modulated by many approaches including multi-layered microparticles, nanoparticles-in-microparticles, hydrogel templates, coaxial electrospray and microfluidic fabrication, etc. (Han et al., 2016a) Interestingly, the initial burst can be advantageous to produce pulsatile-release MPs that mimic the prime-boost effect of conventional vaccines, showing the potential to make single-injection vaccines (Guarecuco et al., 2018). After administration, the bulk degrading PLGA MPs can generate acidic degradation products which may affect both antigen stability and release kinetics (McHugh et al., 2015). These pH issues may be alleviated by preparation of small PLGA particles which facilitate the diffusion of acidic degradation products and by addition of insoluble buffers to maintain a stable interior pH (McHugh et al., 2015).

### Scaffolds

**FIGURE 3:**
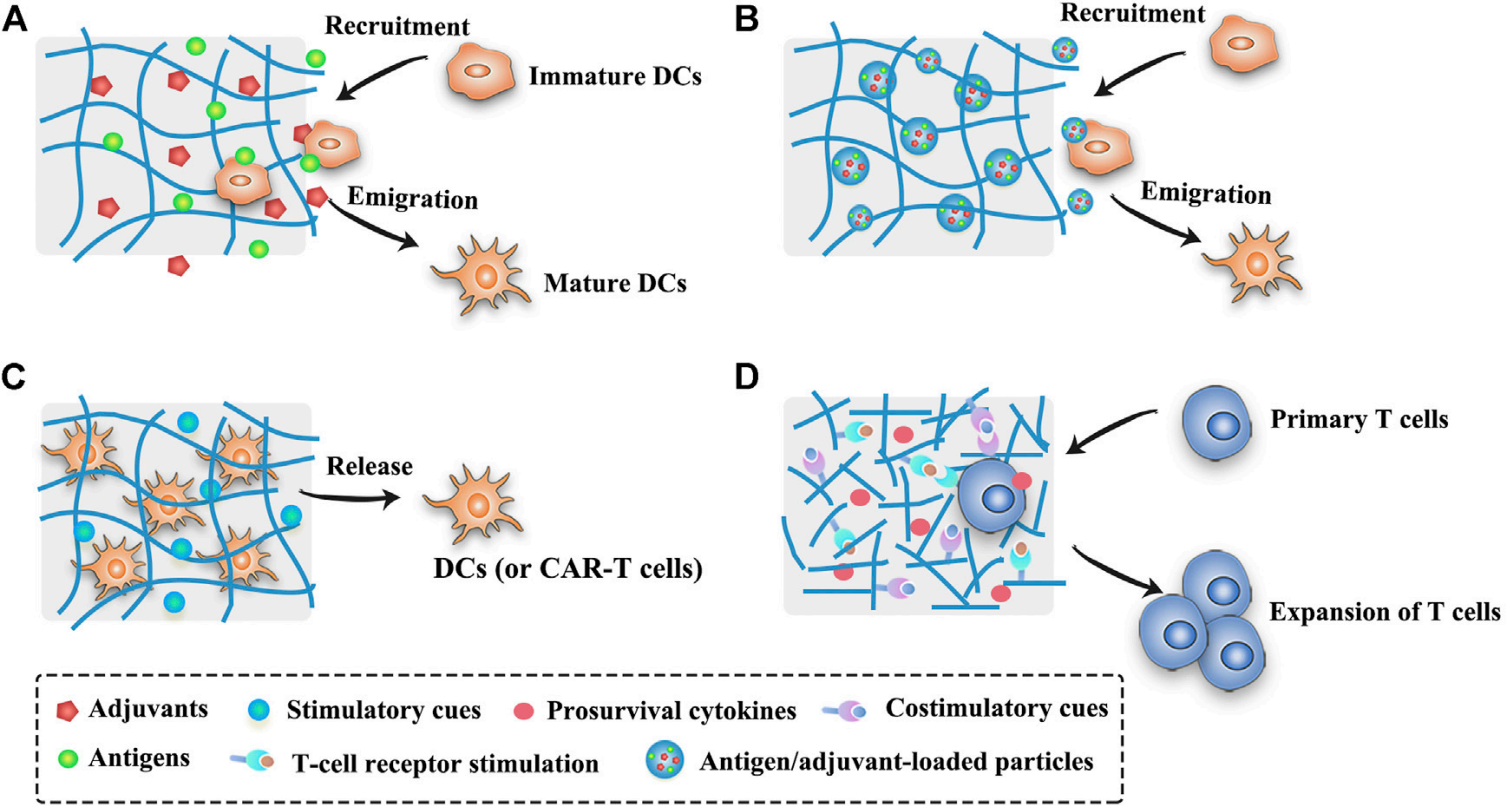
Schematic illustration of strategies of scaffold- or hydrogel-based cancer vaccines. **(A)** Scaffold- or hydrogel-based cancer vaccines can recruit immature DCs into the injection site and subsequently activate them into mature DCs. **(B)** Nanocomposite hydrogel/scaffold systems can co-deliver antigens and adjuvants into the same DCs. **(C)** Scaffold- or hydrogel-based adoptive cell transfer can release mature DCs or CAR-T cells in a sustained manner and maintain the viability of cells. **(D)** Functionalized scaffolds can mimic APCs to directly expand primary T cells.

The scaffold system is commonly used in tissue engineering as the artificial extracellular matrix for the proliferation and differentiation of seeded cells or as the sustained delivery vehicles for therapeutic substances (Bessa et al., 2008; Shafiee and Atala, 2017). Nowadays, scaffolds or implants are also implemented in the field of cancer therapy (Table 2), providing new insights into the design of cancer vaccines. In order to modulate the antitumor immunity, scaffolds can be surgically implanted or low-invasively injected into the body to form localized immune niches or reservoirs for the controlled delivery of engineered immunocytes or cancer vaccines (Figures 3A–C). Various organic or inorganic materials can be used to fabricate scaffolds, such as alginate, collagen, hyaluronic acid, PLGA, and silica rods (Ali et al., 2009b; Kim et al., 2015; Stephan et al., 2015; Phuengkham et al., 2018). Early implants require surgical procedures to place the pre-formed scaffolds into the body to concentrate drug release for a long period of time at the target site. These pre-formed implantable scaffolds have a defined size and shape and can be fabricated in various sophisticated and functionalized structures to meet the needs of different medical applications. In addition, they usually exhibit an interconnected porous architecture, facilitating the diffusion of encapsulated immunomodulatory cues and providing space for the expansion and interaction of encapsulated immune cells or the incoming immune cells (Weiden et al., 2018) (Figure 3). Moreover, the release profiles of the biomolecules or immunocytes in the scaffolds can be fine-tuned to modulate the immune cell function (Ali et al., 2011b; Weiden et al., 2018). The pioneering work of scaffold vaccine was done by the Mooney group, who designed macroporous PLGA scaffolds. These scaffolds released GM-CSF in a sustained manner to recruit host DCs and subsequently presented tumor cell lysates and CpG to activate the incoming DCs (Ali et al., 2009a; Ali et al., 2009b). Implantation of this scaffold vaccine generated strong tumor-specific T cell responses, resulting in significant tumor regression and survival improvement in both melanoma and glioma mouse models (Ali et al., 2009a; Ali et al., 2009b; Ali et al., 2011a). Various chemokines, immunoadjuvants and cytokines can be also incorporated into this flexible scaffold to direct the recruitment of DC subset and augment the therapeutic activity in combination with checkpoint antibodies (Ali et al., 2013; Ali et al., 2014; Ali et al., 2016). Encouragingly, a human version of the PLGA scaffold vaccine (designated as WDVAX) is currently being evaluated in a phase I clinical trial for metastatic melanoma (US National Library of Medicine, 2021). Although the implantable scaffolds have the ability to control their structure shape and rigidity as well as prevent the diffusion of scaffold materials, the invasive surgical implantation is quite cumbersome, causing patient discomfort and increasing the infection risk (Li and Mooney, 2016; Weiden et al., 2018). These implanted scaffolds cannot be placed in areas that are surgically inaccessible or volume-sensitive due to their stiffness (Leach et al., 2019). In addition, the function of normal organs may be affected if the scaffolds are placed into the tumor resection bed or near the tumor (Leach et al., 2019).

Moving forward, the injectable scaffolds that gel or self-assemble in the body have presented highly deformable and self-organizing abilities, thus avoiding the risks associated with implant surgery. Mooney et al. designed a fascinating injectable and self-assembling scaffold vaccine which was based on mesoporous silica rods (MSRs) with high-aspect-ratio to reprogram the immune cells and enhance the vaccine efficacy (Kim et al., 2015; Li et al., 2018). Upon subcutaneous injection in mice, these MSRs spontaneously assembled themselves into three-dimensional (3D) macroporous structures for the influx and efflux of immune cells. Analogous to previous studies, this injectable inorganic scaffold extended the release of the embedded GM-CSF, CpG and model antigens to recruit and educate host immune cells, leading to significant production of systemic antibodies and cytotoxic T cells that effectively delayed the tumor growth (Kim et al., 2015). The adsorption of cationic polymer polyethyleneimine (PEI) in the previous MSR vaccine could greatly enhance the immunogenicity of neoantigens and eradicate three different established tumors (Li et al., 2018). Besides, surface modifications with poly(ethylene glycol) (PEG) or integrin-binding ligand RGD on MSR scaffolds could also regulate immune cell infiltration and activation (Li W. A. et al., 2016). Notably, these MSR scaffolds were demonstrated to be a promising platform for the delivery of DNA-based cancer vaccines (Nguyen et al., 2020). Different to those macroscopic scaffolds aiming to reprogram the incoming DCs, scaffolds that were composed of lipid bilayer-coated micro-MSRs and functionalized with T cell activation cues (anti-CD3, anti-CD28 and IL-2) could mimic APCs to directly expand primary T cells (Cheung et al., 2018) (Figure 3D). These inorganic scaffolds showed great potential in the delivery of personalized cancer vaccines, however, the biocompatibility, biodegradability and safety of injected materials need to be further evaluated before their clinical use. Other administration methods apart from surgical implantation and syringe injection are waited to be explored to broaden the application of controlled-release vaccines. In an example of this, an *in situ* formed fibrin gel was sprayed into the post-surgery tumor site to control the release of therapeutic antibodies (Chen et al., 2019b). In addition to these PLGA and MSR scaffolds, injectable biomaterials including hydrogels, cryogels, and other *in situ* forming platforms can also form the porous matrix *in vivo* to modulate the immune system. The opportunities and challenges of these injectable biomaterials are described below.

### Injectable Gels

Injectable gels are widely applied in many areas including wound healing, bone regeneration, diabetes treatment and cosmetic surgery (Li et al., 2021). Recently, macroscopic injectable gels, especially intelligent hydrogels with the size ranging from millimetres to centimetres, have attracted enormous attention as biocompatible carriers for the spatiotemporal control over cancer vaccines involving antigens, immunomodulators and engineered immunocytes (Table 2). Injectable gels are flowable fluid before or at the time of injection. Once injected into the body, they immediately undergo “sol-to-gel” phase transition by means of chemical or physical crosslink (Li and Mooney, 2016), loss of shear force (Shear-thinning hydrogels) (Yan et al., 2010), mechanical collapse and recover (such as cryogels) (Bencherif et al., 2015), or solvent exchange (such as phospholipid-based gels) (Han et al., 2016b) to form gels. Consequently, injectable gels can be localized almost anywhere in the body by a syringe without the complicated surgical procedures of implantable scaffolds. More importantly, injectable gels have a highly deformable ability to fit the spatial structure of the injection site before they form into a persisting implant (Leach et al., 2019).

Hydrogels are crosslinked 3D polymeric networks containing a large amount of water (typically 70–99%) which enables them to mimic the extracellular matrix (Li and Mooney, 2016). Due to the excellent biocompatibility and tunable release kinetics, hydrogels are the most extensively explored gel carriers in the delivery of cancer vaccines. In addition, hydrogels have high loading capacity for hydrophilic drugs, and they are harmless to the integrity of antigens because the encapsulation process is usually carried out under mild aqueous conditions. Moreover, antigens can be protected in the hydrogels from degradation by the *in vivo* enzymes during the extended periods of antigen presentation. Hydrogels can be made from a variety of materials including peptides (Wang et al., 2018; Wang Z. et al., 2020), proteins (e.g., silk fibroin and gelatin) (Wu et al., 2016; Oh et al., 2017), polysaccharides (e.g., alginate, hyaluronic acid and chitosan) (Hori et al., 2009; Kordalivand et al., 2019; Ye et al., 2019), nucleic acids (e.g., DNA) (Li et al., 2020) and synthetic polymers (e.g., poloxamers and polyesters) (Cirillo et al., 2019; Leach et al., 2019). To form hydrogels, these materials can be crosslinked through different mechanisms, mainly by physical noncovalent interactions (such as hydrogen bonding, host-guest interactions, hydrophobic interactions, electrostatic interactions, etc.) and by chemical covalent bonds (such as click chemistry, Diels-Alder reactions, Michael addition, enzymatic reactions, etc.) (Yu and Ding, 2008; Li and Mooney, 2016; Lei and Tang, 2019).

Many macroscopic hydrogels seek to recruit and activate the endogenous DCs by prolonging the presentation of the antigens and immunomodulators (Figure 3A) (Bencherif et al., 2015; Ye et al., 2019; Duong et al., 2020a). In one representative study, irradiated tumor cells, GM-CSF and CpG were co-encapsulated in sponge-like macroporous cryogels which were fabricated by alginate with RGD peptide modification (Bencherif et al., 2015). After subcutaneously injected into the mice bearing melanoma, these cyrogels released the immunomodulators in a sustained and localized manner to facilitate the influx of DCs and subsequently induce robust and durable tumor-specific T cell responses. In addition, hydrogels are excellent delivery vehicles for adoptive cell therapies (such as DC vaccines and CAR-T cells, Figure 3C) due to the concentration of the engineered immunocytes at the desired site as well as the maintenance of cell viability for a long time (Hori et al., 2009; Yang et al., 2018). Furthermore, hydrogels can easily co-deliver diverse drugs using one platform for a combination of cancer treatments (Wang et al., 2018; Song et al., 2019; Wang et al., 2020b). For example, Wang et al. developed a personalized cancer vaccine (termed PVAX) by integrating JQ1 (an inhibitor of PD-L1 expression) and indocyanine green (ICG, a photosensitizer) co-loaded autologous tumor cells into a hydrogel matrix composed of tumor-penetrable peptides (Wang et al., 2018). After intratumoral administration, the PVAX was triggered by near-infrared (NIR) irradiation to release tumor antigens and JQ1, causing the full activation of the postoperative antitumor immunity that efficiently prevented tumor recurrence and metastasis.

Numerous studies are striving to develop methods for on-demand control over the release kinetics of injectable gels to improve the spatiotemporal delivery of cancer vaccines. Various intelligent hydrogels have been designed to respond to intrinsic or extrinsic stimuli such as low pH, temperature, enzymes, redox, photoirradiation, magnetic fields and ultrasound (Lu et al., 2017; Kanwar and Sinha, 2019). Duong et al. developed an injectable thermosensitive smart hydrogel by conjugating thermo-responsive poly(ε-caprolactone-co-lactide) ester and levodopa to hyaluronic acid (Duong et al., 2020a). This hydrogel exhibited sol-to-gel transition in response to the body temperature, and the levodopa moieties were introduced to strengthen the stability of the hydrogel during implantation. The sustained degradation of this smart hydrogel led to a controlled release of GM-CSF and nano-sized polyplexes expressing model protein antigen ovalbumin (OVA), and then effective inhibition of B16/OVA melanoma tumors by a single injection was observed. Furthermore, many dual-sensitive hydrogels have gained enormous attention in more precisely controlling the release profiles of drugs, such as thermo-pH dual-sensitive hydrogels (Liu et al., 2019a), thermo-reactive oxygen species (ROS) dual-sensitive hydrogels (Yu et al., 2018) and electric field-pH double-sensitive hydrogels (Qu et al., 2018). Interestingly, Brudno et al. developed a replenishable and injectable hydrogel depot system which could capture systemically administrated prodrug refills and then release them locally in a sustained manner (Brudno et al., 2018). This repeatedly refillable hydrogel can be used to prepare precisely controlled-release or pulsatile-release cancer vaccines.

The lag time during the sol to gel transformation may induce a severe initial burst release effect which is responsible for the toxic side effects of immunomodulators and even immune tolerance (Wang and Mooney, 2018; Kanwar and Sinha, 2019). Many strategies are conceived to settle this issue, for example, burst release can be alleviated by optimizing the molecular weight and the concentration of polymers, by using appropriate solvents, and by adding plasticizers or surfactants (Kanwar and Sinha, 2019). The prompt interactions of antigens (and adjuvants) with polymers can also regulate the release profiles, such as by electrostatic interaction or covalent binding (Umeki et al., 2015; Yu et al., 2018; Kordalivand et al., 2019). In one representative study, cationic OVA or cationic OVA peptide were bound to anionic CpG DNA hydrogel by electrostatic interaction, resulting in significantly slower drug release and better tumor inhibition in the group of cationic OVA hydrogel than the group of unmodified OVA hydrogel (Umeki et al., 2015). Another strategy to mitigate the burst effect is to use nanocomposite hydrogel systems (Figure 3B). For example, the incorporation of OVA and two adjuvants (MPLA and Quil A)-loaded PLGA nanoparticles into thermoresponsive pentablock copolymer (PEG-PCL-PLA-PCL-PEG) hydrogels could greatly reduce the burst release of antigens and adjuvants (Bobbala et al., 2016). In another study, OVA was encapsulated into cationic chitosan nanoparticles and then incorporated in hyaluronic acid-based hydrogels in order to achieve a prime-boost regimen (Korupalli et al., 2019). This nanocomposite hydrogel system could rapidly release the nanoparticles that had no specific interactions with the hydrogels for a priming dose, and retain the nanoparticles that were bonded to the hydrogels by covalent or electrostatic interactions for a booster dose. With the minimal burst effect, these chitosan nanoparticles could promote antigen uptake and DC activation, thus inducing higher antibody responses than soluble antigens. Moreover, an onion-structure multilayer hydrogel capsules may be another promising approach to effectively inhibit the burst release of therapeutic cargos (Zhang et al., 2019).

Despite these encouraging progresses, there still exist many hurdles lying on the road to the broad clinical application of hydrogels, including safety concerns about the elaborate materials and solvents, the difficulty in the highly precise control of the release kinetics, the inconsistent drug release resulted from the inconsistent shape of the gels formed, the challenge of *in situ* injectability to deep tumors, the complexity of scaling-up manufacturing, and the difficulty in storage and terminal sterilization (Kanwar and Sinha, 2019). Consequently, the influence of the polymer composition, gel stiffness, network density, porosity and drug-polymer interactions on the release kinetics, as well as the influence of combination therapies, drug doses, administration routes and times on the immune system, should be better understood and these factors should be optimized to exert the maximum efficacy of hydrogel-based cancer vaccines.

### Microneedles

Most vaccines are administrated *via* intramuscular (IM), subcutaneous (SC) and intradermal (ID) inoculation using conventional hypodermic needles. Although the relative immunogenicity of vaccines by these three routes (IM, SC and ID) varies among individual vaccines, ID immunization usually induces more robust immune responses than IM or SC immunizations in clinical studies. This is partly because the different skin layers host a tight semi-contiguous network of immune cells that can interact with the antigens administered to direct drastically different immune responses compared to subcutaneous fat and muscle tissue (Al-Zahrani et al., 2012). The development of MNs is a great achievement to take advantage of skin immunization and to address the issues associated with vaccination by conventional needles (e.g., pain, needle-stick injuries or needle re-use) (Leone et al., 2017). MNs, arrays of sharp tiny needles at lengths varying from 100 to 2,000 μm, are designed to pierce the *stratum corneum* of the skin to deliver both small molecular and macromolecular drugs or even nanoparticles (NPs) to the epidermal and the dermal or even deeper layers in a safe and controlled manner (Alimardani et al., 2021). As a painless transcutaneous administration platform, MNs have been extensively explored for cancer vaccines, antiviral immunotherapies, and diabetes (Jin et al., 2018b; Chen et al., 2020b; Uppu et al., 2020). MNs can promote the deposition of the vaccines by creating micropores in the skin, facilitating vaccine transport, and hence reducing the necessary dose of antigens/vaccines for the immunological responses. A significant number of MNs mediated vaccine candidates have shown encouraging results in preclinical and clinical trials (Hossain et al., 2020).

**FIGURE 4:**
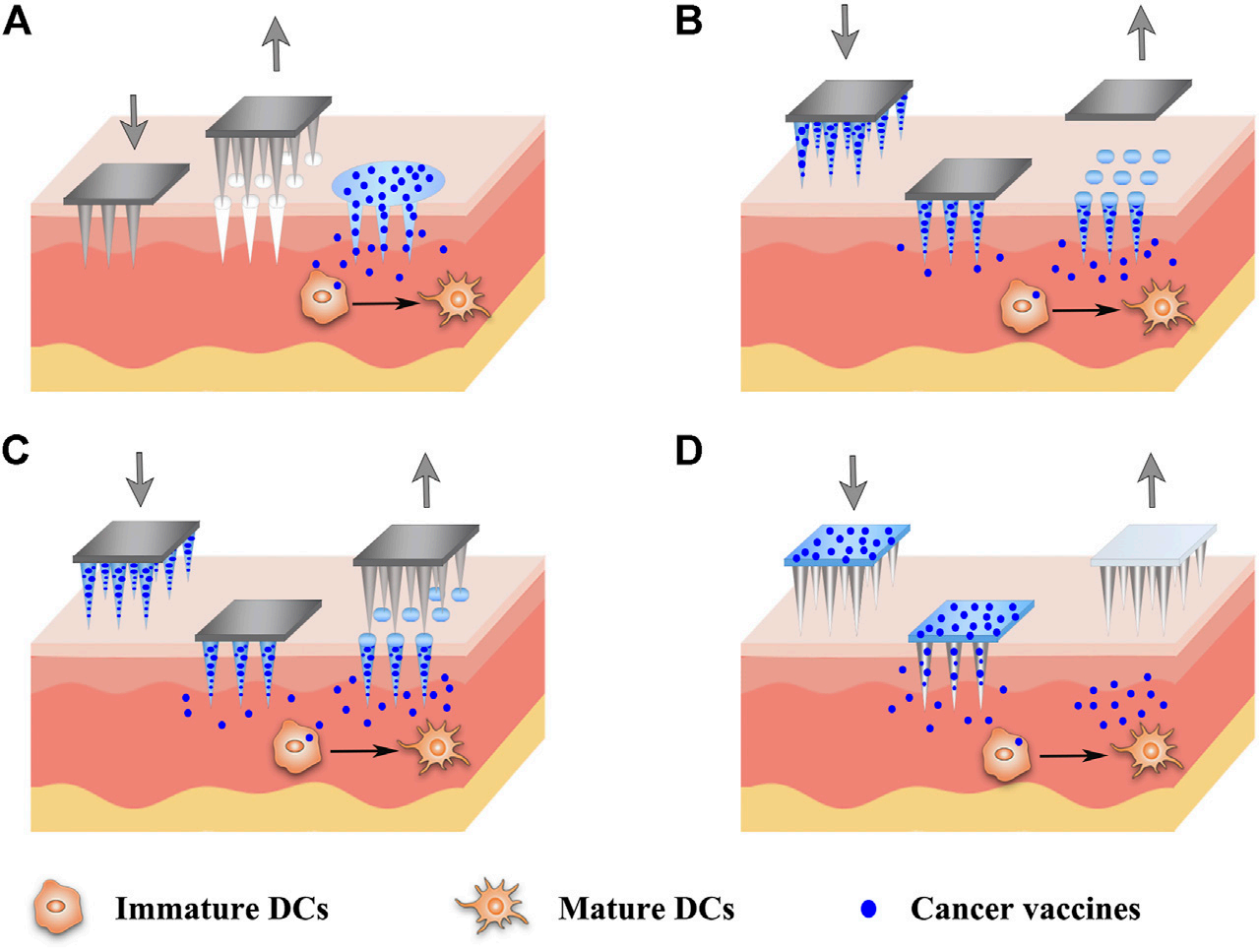
Schematic diagram of strategies of different MN-based cancer vaccines. Various types of MNs have been investigated for the delivery of cancer vaccines, including solid MNs **(A)**, dissolving MNs **(B)**, coated MNs **(C)** and hollow MNs **(D)**.

The minimally invasive and cost-effective features of MNs have aroused their rapid development and extensive exploration in cancer vaccine delivery recently (Table 2). Various types of MNs have been investigated for cancer vaccine delivery, as depicted in Figure 4, including solid MNs (Bhowmik et al., 2011; Chablani et al., 2019), dissolving MNs (Zaric et al., 2013; Gala et al., 2018; Kim et al., 2018; Cole et al., 2019; Kim et al., 2019), coated MNs (Zeng et al., 2017; Duong et al., 2018) and hollow MNs (van der Maaden et al., 2018; Niu et al., 2019). Novel design to utilize parallel circular blades with MN on edge as electrodes was also studied for their ability to deliver siRNA targeting PD-L1 alone, or combined with anti-PD-1 antibody and immunoadjuvant CpG 2395 (Yang et al., 2021). This immunotherapy treatment has been tested in two tumor xenograft murine models and produced robust T cell immune responses and significant tumor growth inhibition as well as excellent safety profiles. Various vaccine formulations, including microparticles (Bhowmik et al., 2011), nanoparticles (Zaric et al., 2013), nanopolyplex (Duong et al., 2020b), liposomes (van der Maaden et al., 2018), and *in situ* generating nanomicelles (Kim et al., 2018) to ensure efficient co-encapsulation of antigens and adjuvants within the nanovaccine platforms have been delivered using MNs to improve cancer vaccine therapy. Du et al. used hollow MNs to administer a range of nanoparticle vaccine formulations loaded with OVA and poly(I:C) (Du et al., 2017). PLGA nanoparticles, liposomes, mesoporous silica nanoparticles, and gelatin nanoparticles were compared. The co-encapsulation of OVA and poly(I:C) using those formulations yielded a similar IgG1 response, but a surprisingly higher IgG2a response than OVA/poly(I:C) solution in a murine model. Particularly, PLGA nanoparticles and especially cationic liposomes, which presented sustained release profiles of both OVA and poly(I:C), elicited better immune responses (Du et al., 2017). The antigens and adjuvants were co-encapsulated in some vaccine formulations through electrostatic interaction, hydrophobic interaction as well as covalent linking interaction (Chen et al., 2021). However, when these formulations are loaded into the MNs, especially adding some excipients to form the MNs, the electrostatic interaction between the antigen and adjuvant is hampered. As a good example to address this issue, an *in situ* generating nanomicelle including hydrophilic tumor model antigen (OVA), hydrophobic adjuvant TLR 7/8 agonist (R848) and amphiphilic triblock copolymer has been delivered using dissolving MNs platform to form nanomicelles *in situ* with a size of 30–40 nm after cutaneous application (Kim et al., 2018). Antigen-specific humoral and cellular immunity protection were found to be elicited after administration, leading to a significant antitumor activity in the EG7-OVA tumor-xenograft mice. Since MNs are usually applied topically on the surface of the skin, they are very suitable to be combined with phototherapy (Ye et al., 2017; Chen et al., 2020a). One example of such combination therapies was based on the tumor lysates, melanin and GM-CSF were loaded into hyaluronic acid-based MNs that allowed sustained release of the lysates (Ye et al., 2017). After administration, the melanin converted the NIR irradiation into heat, which facilitated tumor-antigen uptake by DCs and boosted the antitumor immune responses in both B16F10 and 4T1 tumor models.

Collectively, MNs have offered a unique set of properties for cancer vaccine delivery with improved vaccination efficacy, compliance, and coverage. The use of MNs instead of a needle injection can not only avoid pain for the patients but also provide the promise of self-vaccination as well as release the assistance from professional healthcare, which is especially suitable for mass vaccination in the case of a pandemic (Zaric et al., 2013). Although the use of MNs could be more effective than a single-needle injection as the antigens might be more evenly distributed after injection and have a more targeted delivery to APCs in the dermis and epidermis layers under the skin, MNs-based delivery may cause more frequent local reactions due to the shallow penetration (Zhang et al., 2015). MNs can be fabricated in different shapes using various materials, including stainless steel, titanium, silicon, ceramic and polymers (Alimardani et al., 2021). However, there is a possibility of needle breakage within the skin after the application of metal, silicon or glass MNs, triggering unintentional safety concerns (Jin et al., 2018a). In addition, some materials such as nickel may cause allergic reactions. Biodegradable polymeric MNs can minimize the risk of inflammation induced by undissolving materials, because they can degrade to metabolizable objects in the body (Jin et al., 2018a). On the other hand, MNs should have enough mechanical strength and sharpness to pierce the skin for transdermal applications. However, the incorporation of drugs and nanoparticles may compromise the mechanical strength of MNs (Kim et al., 2018). Therefore, the design of MNs-based cancer vaccines should be optimized with the full consideration of balancing the pain, mechanical strength, drug doses and drug release profiles for therapeutic efficacy. For a cancer vaccine, the efficacy is closely associated with the distance from the injection site to the cancer site. Most of the studies for MN-based cancer vaccines were conducted in malignant melanoma. Although they can provide invaluable information about other cancers as well, more cancer models will need testing for evaluating the systemic immune protections of MNs (Zhang et al., 2015).

## Correlations of the Immune Responses and the Release Kinetics of Cancer Vaccines

It has been widely reported that controlled or sustained release of antigens and adjuvants is beneficial to generate antigen-specific antibodies, T cell responses and immunological memory responses (Kanchan et al., 2009; Ali et al., 2011b; Demento et al., 2012; Hanet al., 2016b; Irvine et al., 2020). The mechanisms underlying these benefits of controlled-release vaccines may be complex and multifactorial. Herein, we describe four major mechanisms as follows. Firstly, controlled-release platforms usually form an *in situ* drug depot in which antigens can maintain their intact conformation for a considerable period of time. Antigens and adjuvants can be released in a continuous or pulsatile manner to extend the antigen exposure or to mimic the traditional prime-boost regimen of vaccination (Sivakumar et al., 2011; Brito and O'Hagan, 2014). Secondly, owing to the heterogeneity and immunostimulatory activity of materials or the effect of encapsulated chemokines, most depot-like controlled-release systems can stimulate the accumulation of inflammatory cells or recruit APCs to the injection sites (Ali et al., 2009b; Brito and O'Hagan, 2014; Han et al., 2016b). Subsequently, cytokines secreted by inflammatory cells can stimulate the activation, proliferation and differentiation of immune cells. On the other hand, the antigen depot is surrounded or infiltrated by the recruited APCs, thus increasing the chance of antigen uptake. Thirdly, controlled-release systems, especially when loaded with immunoadjuvants, can further motivate the maturation of APCs and then direct the Th1 or Th2 immune responses (Li et al., 2018; Song et al., 2018). Last, but not least, some polymeric micro- or nanoparticles mimicking the size and surface characteristics of pathogens or functionalized with targeting ligands can accumulate in lymph nodes or target the specific immunocytes (Mata et al., 2011; Silva et al., 2016), thereby reducing the antigen dose and improving the magnitude of immune responses.

Although controlled-release systems have the potential to augment the vaccine efficacy with minimal toxicity, the effect of release kinetics on immune responses is complex and unclear. It is worth noting that antigen stimulation can be either immunologically activating or immunologically tolerant. For example, intravenous injection of low-dose antigens could induce immune activation, whereas intravenous injection of high-dose antigens might cause T cell exhaustion (Zinkernagel et al., 1997). Although the slow and sustained release of vaccines is demonstrated to favor the antitumor immunity, the extremely long periods of antigen presentation may, in turn, hamper the antitumor immunity by exhausting and deleting tumor-specific T cells or directing T cells to the antigen depot rather than to the target tumor (Iezzi et al., 1998; Hailemichael et al., 2013; Shae et al., 2019). On the other hand, a particularly fast release of antigens may result in rapid clearance of antigens from the lymph nodes (Shae et al., 2019; Irvine et al., 2020), necessitating repeated injections to boost the immune responses. Moreover, the release rate of adjuvants seems necessary to be coincident with that of antigens, because a fast release of adjuvants leads to early exhaustion of adjuvants, while an extremely slow release of adjuvants cannot provide sufficient activation cues to the immature DCs, thereby promoting T cell anergy and immune tolerance (Audiger et al., 2017; Wang and Mooney, 2018; Leach et al., 2019). Therefore, the release kinetics of antigens and adjuvants from the controlled-release platforms has a significant influence on the type and magnitude of immune responses. An interesting study showed that a “dose escalation” regimen with an exponentially increasing antigen dose elicited a superior CD8^+^ T cell response than a single bolus injection or constantly repeated dose regimen (Johansen et al., 2008). Another opinion suggested that an appropriate burst release followed by a sustained vaccine release was optimal to induce fast and powerful immune responses (Zhang et al., 2014). Consequently, a highly precisely controlled vaccine delivery system with on-demand drug release is believed to maximize the immune efficacy. Nevertheless, the optimal vaccine release kinetics for inducing the most potent and durable immunity is still not clear. It may vary among different types of controlled-release vaccines which have distinct adjuvant properties, antigen types, disease types and administration routes (Brito and O'Hagan, 2014; McHugh et al., 2015). Moreover, antigens and adjuvants can hardly share consistent release profiles due to their different physicochemical properties. Therefore, The release kinetics of antigens and adjuvants should be screened and optimized to augment the antitumor immunity. Fortunately, the release kinetics can be modulated by numerous factors, including material properties (such as chemical composition, molecular weight, hydrophobicity, crystallinity, glass transition temperature and degradation products), depot geometry (such as size, shape, porosity and network density), antigenic factors (such as antigen size, position and loading amount), and others (such as antigen-polymer interactions, additives and administration route) (McHugh et al., 2015; Li and Mooney, 2016). Furthermore, strategies that guarantee the simultaneous presentation of antigens and adjuvants to the same APCs during the long period of release may exert stronger antigen-specific CD8^+^ T cell responses. For example, antigens and adjuvants can be linked together by covalent or ionic bonds before loaded into controlled-release platforms, or antigens and adjuvants can be pre-encapsulated into nano- or microparticles followed by incorporating these particles into a macroscopic matrix.

Depiction of the drug release profiles is the inevitable step to study the controlled-release vaccines. However, most studies contain only the *in vitro* release data, which may not be coincident with the *in vivo* release due to the different release environment inside and outside the body. Unfortunately, the accurate quantitative data of *in vivo* release kinetics are difficult to acquire owing to the low antigen loading amount and long duration of release (McHugh et al., 2015). *In vivo* imaging is now commonly used to semi-quantitate the *in vivo* vaccine kinetics by measuring the remaining fluorescence intensity of fluorescently labeled antigens in the vaccine depot over time. However, it is unable to determine the actual amount and stability of the antigens because fluorescence quenches easily. Therefore, both the exploration of more accurate *in vivo* measuring technologies and the in-depth understanding of *in vitro-in vivo* correlations are needed to spur the development of controlled-release vaccines. By far, many controlled-release vaccine delivery systems have been using model antigens (such as OVA) and model tumors (such as immortalized tumor cell lines) to study the release kinetics and antitumor immune responses (Kim et al., 2015; Umeki et al., 2015; Han et al., 2016b; Niu et al., 2019). However, these model antigens and the model tumors cannot recapitulate the complex challenges facing the real therapeutic antigens and patient individualized tumors (Day et al., 2015; McHugh et al., 2015). For example, the model antigen ovalbumin for mouse studies is exceptionally immunogenic and may lead to overestimating vaccine efficacy. Moreover, subcutaneous injection of B16F10 cell lines into the mice is widely used to establish melanoma, but these *in vitro* cultured murine cancer cells cannot simulate the cancer heterogeneity and are not physiologically relevant to human cancers. Moreover, the immune system of a healthy mouse differs significantly from that of a human in both innate and adaptive immunity (Mestas and Hughes, 2004). Therefore, more data on the immune efficacy of controlled-release vaccines that are tested using clinically relevant antigens and using suitable tumor and animal models should be assessed in the future.

## Conclusions and Outlook

Cancer vaccines represent the most promising and cost-effective antitumor strategy which engages the immunity of patients to target malignancies. Depending on the form and source of antigens, cancer vaccines can be briefly categorized into five types, including subunit vaccines, genetic vaccines, DC-based vaccines, tumor cell-based vaccines and *in situ* cancer vaccines. The pros and cons of these vaccines have been outlined in this review. Local and sustained delivery of vaccines at lower doses shows higher effectiveness and lower toxicity than bolus injections. Recently, the controlled-release drug delivery systems, such as polymeric MPs, scaffolds, hydrogels and MNs, have attracted enormous attention in the spatiotemporal delivery of cancer vaccines. However, there are still many obstacles limiting the broad application of controlled-release cancer vaccines, including the instability of antigens and adjuvants during the preparation and release period, the challenge of precisely controlling over release kinetics, safety concerns, and the difficulties in scaling-up manufacturing, storage and terminal sterilization. The mechanisms and patterns of the action of controlled-release vaccines need to be studied in-depth to develop potent, persistent, non-toxic and easily available single-shot cancer vaccines.

Despite the fact that immunotherapy have been widely studied and applied on hematological tumors and melanoma, the effect of current immunotherapies on solid tumors remains faint because of the immunosuppressive tumor microenvironments, low tumor immunogenicity and low infiltration of immune cells into tumors (Kuol et al., 2017; Galluzzi et al., 2018). Based on the unique advantages of these controlled-release systems as mentioned above, the combination of multiple cancer therapies with controlled-release cancer vaccines may be the most promising approach to treat solid tumors as well as to prevent tumor metastasis and recurrence. For example, many clinical available strategies, such as chemotherapy, radiotherapy, phototherapy, oncolytic viruses and immune checkpoint antibodies, show great therapeutic potential in combination with cancer vaccines. Moreover, targeting the microbiome in the intestine or the tumor may have a synergistic antitumor effect with cancer vaccines (Matson et al., 2018; Nejman et al., 2020). More work should be done to explore the synergistic therapeutic effects of controlled-release vaccines and other therapies. In conclusion, the next generation of cancer vaccines should focus on the following aspects: 1) the choice of antigens with high immunogenicity; 2) co-delivering antigens and appropriate adjuvants to the same APCs; 3) modulating the optimal release kinetics of the antigens and adjuvants from delivery systems to augment subsequent immune responses; 4) reshaping the immunosuppressive environment to maximize the specific cytotoxic function of T cells; 5) adopting optimal combinatorial therapeutic regimens to improve the outcomes and avoid tumor evade.

## References

[B1] Al-Zahrani S. Zaric M. McCrudden C. Scott C. Kissenpfennig A. Donnelly R. F. (2012). Microneedle-mediated Vaccine Delivery: Harnessing Cutaneous Immunobiology to Improve Efficacy. Expert Opin. Drug Deliv. 9 (5), 541–550. 10.1517/17425247.2012.676038 22475249 PMC4119955

[B2] Ali O. A. Doherty E. Bell W. J. Fradet T. Hudak J. Laliberte M.-T. (2011a). The Efficacy of Intracranial PLG-Based Vaccines Is Dependent on Direct Implantation into Brain Tissue. J. Controlled Release 154 (3), 249–257. 10.1016/j.jconrel.2011.06.021 21704093

[B3] Ali O. A. Doherty E. Mooney D. J. Emerich D. (2011b). Relationship of Vaccine Efficacy to the Kinetics of DC and T-Cell Responses Induced by PLG-Based Cancer Vaccines. Biomatter 1 (1), 66–75. 10.4161/biom.1.1.16277 23507728 PMC3548245

[B4] Ali O. A. Emerich D. Dranoff G. Mooney D. J. (2009a). *In situ* regulation of DC Subsets and T Cells Mediates Tumor Regression in Mice. Sci. Translational Med. 1 (8), 8ra19. 10.1126/scitranslmed.3000359 PMC2872791 20368186

[B5] Ali O. A. Huebsch N. Cao L. Dranoff G. Mooney D. J. (2009b). Infection-mimicking Materials to Program Dendritic Cells In Situ. Nat. Mater 8 (2), 151–158. 10.1038/nmat2357 19136947 PMC2684978

[B6] Ali O. A. Lewin S. A. Dranoff G. Mooney D. J. (2016). Vaccines Combined with Immune Checkpoint Antibodies Promote Cytotoxic T-Cell Activity and Tumor Eradication. Cancer Immunol. Res. 4 (2), 95–100. 10.1158/2326-6066.Cir-14-0126 26669718 PMC4740221

[B7] Ali O. A. Tayalia P. Shvartsman D. Lewin S. Mooney D. J. (2013). Inflammatory Cytokines Presented from Polymer Matrices Differentially Generate and Activate DCs In Situ. Adv. Funct. Mater. 23 (36), 4621–4628. 10.1002/adfm.201203859 24688455 PMC3968866

[B8] Ali O. A. Verbeke C. Johnson C. Sands R. W. Lewin S. A. White D. (2014). Identification of Immune Factors Regulating Antitumor Immunity Using Polymeric Vaccines with Multiple Adjuvants. Cancer Res. 74 (6), 1670–1681. 10.1158/0008-5472.CAN-13-0777 24480625 PMC3959905

[B9] Alimardani V. Abolmaali S. S. Tamaddon A. M. Ashfaq M. (2021). Recent Advances on Microneedle Arrays-Mediated Technology in Cancer Diagnosis and Therapy. Drug Deliv. Transl. Res. 11, 788–816. 10.1007/s13346-020-00819-z 32740799

[B10] Allison J. P. (2015). Immune Checkpoint Blockade in Cancer Therapy. JAMA 314 (11), 1113–1114. 10.1001/jama.2015.11929 26348357

[B11] Audiger C. Rahman M. J. Yun T. J. Tarbell K. V. Lesage S. (2017). The Importance of Dendritic Cells in Maintaining Immune Tolerance. J.I. 198 (6), 2223–2231. 10.4049/jimmunol.1601629 PMC5343761 28264998

[B12] Bailey B. A. Ochyl L. J. Schwendeman S. P. Moon J. J. (2017). Toward a Single‐Dose Vaccination Strategy with Self‐Encapsulating PLGA Microspheres. Adv. Healthc. Mater. 6 (12), 1601418. 10.1002/adhm.201601418 PMC5483999 28371568

[B13] Bencherif S. A. Warren Sands R. Ali O. A. Li W. A. Lewin S. A. Braschler T. M. (2015). Injectable Cryogel-Based Whole-Cell Cancer Vaccines. Nat. Commun. 6, 7556. 10.1038/ncomms8556 26265369 PMC4763944

[B14] Bessa P. C. Casal M. Reis R. L. (2008). Bone Morphogenetic Proteins in Tissue Engineering: the Road from Laboratory to Clinic, Part II (BMP Delivery). J. Tissue Eng. Regen. Med. 2 (2-3), 81–96. 10.1002/term.74 18383454

[B15] Bhowmik T. D’Souza B. Shashidharamurthy R. Oettinger C. Selvaraj P. D’Souza M. J. (2011). A Novel Microparticulate Vaccine for Melanoma Cancer Using Transdermal Delivery. J. Microencapsulation 28 (4), 294–300. 10.3109/02652048.2011.559287 21545320

[B16] Bobbala S. Tamboli V. McDowell A. Mitra A. K. Hook S. (2016). Novel Injectable Pentablock Copolymer Based Thermoresponsive Hydrogels for Sustained Release Vaccines. AAPS J. 18 (1), 261–269. 10.1208/s12248-015-9843-4 26589309 PMC4706273

[B17] Bol K. F. Schreibelt G. Gerritsen W. R. de Vries I. J. M. Figdor C. G. (2016). Dendritic Cell-Based Immunotherapy: State of the Art and beyond. Clin. Cancer Res. 22 (8), 1897–1906. 10.1158/1078-0432.CCR-15-1399 27084743

[B18] Bouzid R. Peppelenbosch M. Buschow S. I. (2020). Opportunities for Conventional and In Situ Cancer Vaccine Strategies and Combination with Immunotherapy for Gastrointestinal Cancers, a Review. Cancers 12 (5), 1121. 10.3390/cancers12051121 PMC7281593 32365838

[B19] Brito L. A. O'Hagan D. T. (2014). Designing and Building the Next Generation of Improved Vaccine Adjuvants. J. Controlled Release 190, 563–579. 10.1016/j.jconrel.2014.06.027 24998942

[B20] Browning M. J. (2013). Antigen Presenting Cell/Tumor Cell Fusion Vaccines for Cancer Immunotherapy. Hum. Vaccin. Immunother. 9 (7), 1545–1548. 10.4161/hv.24235 23475129 PMC3890217

[B21] Brudno Y. Pezone M. J. Snyder T. K. Uzun O. Moody C. T. Aizenberg M. (2018). Replenishable Drug Depot to Combat Post-resection Cancer Recurrence. Biomaterials 178, 373–382. 10.1016/j.biomaterials.2018.05.005 29779862 PMC6075722

[B22] Calmeiro J. Carrascal M. Gomes C. Falcão A. Cruz M. T. Neves B. M. (2019). Biomaterial-based Platforms for In Situ Dendritic Cell Programming and Their Use in Antitumor Immunotherapy. J. Immunotherapy Cancer 7 (1), 238. 10.1186/s40425-019-0716-8 PMC6727507 31484548

[B23] Chablani L. Tawde S. A. Akalkotkar A. D’Souza M. J. (2019). Evaluation of a Particulate Breast Cancer Vaccine Delivered via Skin. AAPS J. 21 (2), 12. 10.1208/s12248-018-0285-7 30604321

[B24] Chen F. Wang Y. Gao J. Saeed M. Li T. Wang W. (2021). Nanobiomaterial-based Vaccination Immunotherapy of Cancer. Biomaterials 270, 120709. 10.1016/j.biomaterials.2021.120709 33581608

[B25] Chen M. Quan G. Wen T. Yang P. Qin W. Mai H. (2020a). Cold to Hot: Binary Cooperative Microneedle Array-Amplified Photoimmunotherapy for Eliciting Antitumor Immunity and the Abscopal Effect. ACS Appl. Mater. Inter. 12 (29), 32259–32269. 10.1021/acsami.0c05090 32406239

[B26] Chen Q. Chen J. Yang Z. Xu J. Xu L. Liang C. (2019a). Nanoparticle-enhanced Radiotherapy to Trigger Robust Cancer Immunotherapy. Adv. Mater. 31 (10), e1802228. 10.1002/adma.201802228 30663118

[B27] Chen Q. Wang C. Zhang X. Chen G. Hu Q. Li H. (2019b). *In situ* sprayed Bioresponsive Immunotherapeutic Gel for Post-surgical Cancer Treatment. Nat. Nanotech 14 (1), 89–97. 10.1038/s41565-018-0319-4 30531990

[B28] Chen R. Zhang H. Yan J. Bryers J. D. (2018). Scaffold-mediated Delivery for Non-viral mRNA Vaccines. Gene Ther. 25 (8), 556–567. 10.1038/s41434-018-0040-9 30242259 PMC6309225

[B29] Chen S.-X. Ma M. Xue F. Shen S. Chen Q. Kuang Y. (2020b). Construction of Microneedle-Assisted Co-delivery Platform and its Combining Photodynamic/immunotherapy. J. Controlled Release 324, 218–227. 10.1016/j.jconrel.2020.05.006 32387551

[B30] Cheng S. Xu C. Jin Y. Li Y. Zhong C. Ma J. (2020). Artificial Mini Dendritic Cells Boost T Cell-Based Immunotherapy for Ovarian Cancer. Adv. Sci. 7 (7), 1903301. 10.1002/advs.201903301 PMC7141030 32274314

[B31] Cheung A. S. Zhang D. K. Y. Koshy S. T. Mooney D. J. (2018). Scaffolds that Mimic Antigen-Presenting Cells Enable Ex Vivo Expansion of Primary T Cells. Nat. Biotechnol. 36 (2), 160–169. 10.1038/nbt.4047 29334370 PMC5801009

[B32] Chiang C. Coukos G. Kandalaft L. (2015). Whole Tumor Antigen Vaccines: where Are We?. Vaccines 3 (2), 344–372. 10.3390/vaccines3020344 26343191 PMC4494356

[B33] Cho J.-a. Lee Y.-S. Kim S.-H. Ko J.-K. Kim C.-W. (2009). MHC Independent Anti-tumor Immune Responses Induced by Hsp70-Enriched Exosomes Generate Tumor Regression in Murine Models. Cancer Lett. 275 (2), 256–265. 10.1016/j.canlet.2008.10.021 19036499

[B34] Chu Y. Liu Q. Wei J. Liu B. (2018). Personalized Cancer Neoantigen Vaccines Come of Age. Theranostics 8 (15), 4238–4246. 10.7150/thno.24387 30128050 PMC6096398

[B35] Cirillo G. Curcio U. G. Nicoletta M. Iemma F. P. Iemma F. (2019). Injectable Hydrogels for Cancer Therapy over the Last Decade. Pharmaceutics 11 (9), 486. 10.3390/pharmaceutics11090486 PMC6781516 31546921

[B36] Cole G. Ali A. A. McErlean E. Mulholland E. J. Short A. McCrudden C. M. (2019). DNA Vaccination via RALA Nanoparticles in a Microneedle Delivery System Induces a Potent Immune Response against the Endogenous Prostate Cancer Stem Cell Antigen. Acta Biomater. 96, 480–490. 10.1016/j.actbio.2019.07.003 31299353

[B37] Coley W. B. (1991). The Classic. Clin. Orthopaedics Relat. Res. 262, 3–11. 10.1097/00003086-199101000-00002 1984929

[B38] Da Silva C. G. Camps M. G. M. Li T. M. W. Y. Zerrillo L. Löwik C. W. Ossendorp F. (2019). Effective Chemoimmunotherapy by Co-delivery of Doxorubicin and Immune Adjuvants in Biodegradable Nanoparticles. Theranostics 9 (22), 6485–6500. 10.7150/thno.34429 31588231 PMC6771237

[B39] Day C.-P. Merlino G. Van Dyke T. (2015). Preclinical Mouse Cancer Models: a Maze of Opportunities and Challenges. Cell 163 (1), 39–53. 10.1016/j.cell.2015.08.068 26406370 PMC4583714

[B40] Demento S. L. Cui W. Criscione J. M. Stern E. Tulipan J. Kaech S. M. (2012). Role of Sustained Antigen Release from Nanoparticle Vaccines in Shaping the T Cell Memory Phenotype. Biomaterials 33 (19), 4957–4964. 10.1016/j.biomaterials.2012.03.041 22484047 PMC5724530

[B41] Desrichard A. Snyder A. Chan T. A. (2016). Cancer Neoantigens and Applications for Immunotherapy. Clin. Cancer Res. 22 (4), 807–812. 10.1158/1078-0432.CCR-14-3175 26515495

[B42] Du G. Hathout R. M. Nasr M. Nejadnik M. R. Tu J. Koning R. I. (2017). Intradermal Vaccination with Hollow Microneedles: a Comparative Study of Various Protein Antigen and Adjuvant Encapsulated Nanoparticles. J. Controlled Release 266, 109–118. 10.1016/j.jconrel.2017.09.021 28943194

[B43] Duong H. T. T. Thambi T. Yin Y. Kim S. H. Nguyen T. L. Phan V. H. G. (2020a). Degradation-regulated Architecture of Injectable Smart Hydrogels Enhances Humoral Immune Response and Potentiates Antitumor Activity in Human Lung Carcinoma. Biomaterials 230, 119599. 10.1016/j.biomaterials.2019.119599 31718883

[B44] Duong H. T. T. Yin Y. Thambi T. Kim B. S. Jeong J. H. Lee D. S. (2020b). Highly Potent Intradermal Vaccination by an Array of Dissolving Microneedle Polypeptide Cocktails for Cancer Immunotherapy. J. Mater. Chem. B 8 (6), 1171–1181. 10.1039/c9tb02175b 31957761

[B45] Duong H. T. T. Yin Y. Thambi T. Nguyen T. L. Giang Phan V. H. Lee M. S. (2018). Smart Vaccine Delivery Based on Microneedle Arrays Decorated with Ultra-pH-responsive Copolymers for Cancer Immunotherapy. Biomaterials 185, 13–24. 10.1016/j.biomaterials.2018.09.008 30216806

[B46] Fan Y. Kuai R. Xu Y. Ochyl L. J. Irvine D. J. Moon J. J. (2017). Immunogenic Cell Death Amplified by Co-localized Adjuvant Delivery for Cancer Immunotherapy. Nano Lett. 17 (12), 7387–7393. 10.1021/acs.nanolett.7b03218 29144754 PMC5821496

[B47] Fang R. H. Hu C.-M. J. Luk B. T. Gao W. Copp J. A. Tai Y. (2014). Cancer Cell Membrane-Coated Nanoparticles for Anticancer Vaccination and Drug Delivery. Nano Lett. 14 (4), 2181–2188. 10.1021/nl500618u 24673373 PMC3985711

[B48] Fennemann F. L. de Vries I. J. M. Figdor C. G. Verdoes M. (2019). Attacking Tumors from All Sides: Personalized Multiplex Vaccines to Tackle Intratumor Heterogeneity. Front. Immunol. 10, 824–832. 10.3389/fimmu.2019.00824 31040852 PMC6476980

[B49] Gala R. Zaman R. D’Souza M. Zughaier S. (2018). Novel Whole-Cell Inactivated neisseria Gonorrhoeae Microparticles as Vaccine Formulation in Microneedle-Based Transdermal Immunization. Vaccines 6 (3), 60. 10.3390/vaccines6030060 PMC6161099 30181504

[B50] Galluzzi L. Buqué A. Kepp O. Zitvogel L. Kroemer G. (2017). Immunogenic Cell Death in Cancer and Infectious Disease. Nat. Rev. Immunol. 17 (2), 97–111. 10.1038/nri.2016.107 27748397

[B51] Galluzzi L. Chan T. A. Kroemer G. Wolchok J. D. López-Soto A. (2018). The Hallmarks of Successful Anticancer Immunotherapy. Sci. Transl. Med. 10 (459), eaat7807. 10.1126/scitranslmed.aat7807 30232229

[B52] Gan J. Du G. He C. Jiang M. Mou X. Xue J. (2020). Tumor Cell Membrane Enveloped Aluminum Phosphate Nanoparticles for Enhanced Cancer Vaccination. J. Controlled Release 326, 297–309. 10.1016/j.jconrel.2020.07.008 32659330

[B53] Gardner T. Elzey B. Hahn N. M. (2012). Sipuleucel-T (Provenge) Autologous Vaccine Approved for Treatment of Men with Asymptomatic or Minimally Symptomatic Castrate-Resistant Metastatic Prostate Cancer. Hum. Vaccin. Immunother. 8 (4), 534–539. 10.4161/hv.19795 22832254

[B54] Geng Y. Yuan W. Wu F. Chen J. He M. Jin T. (2008). Formulating Erythropoietin-Loaded Sustained-Release PLGA Microspheres without Protein Aggregation. J. Controlled Release 130 (3), 259–265. 10.1016/j.jconrel.2008.06.011 18620011

[B55] Greening D. W. Gopal S. K. Xu R. Simpson R. J. Chen W. (2015). Exosomes and Their Roles in Immune Regulation and Cancer. Semin. Cel Dev. Biol. 40, 72–81. 10.1016/j.semcdb.2015.02.009 25724562

[B56] Guarecuco R. Lu J. McHugh K. J. Norman J. J. Thapa L. S. Lydon E. (2018). Immunogenicity of Pulsatile-Release PLGA Microspheres for Single-Injection Vaccination. Vaccine 36 (22), 3161–3168. 10.1016/j.vaccine.2017.05.094 28625520 PMC5960071

[B57] Gulley J. L. Borre M. Vogelzang N. J. Ng S. Agarwal N. Parker C. C. (2019). Phase III Trial of PROSTVAC in Asymptomatic or Minimally Symptomatic Metastatic Castration-Resistant Prostate Cancer. Jco 37 (13), 1051–1061. 10.1200/jco.18.02031 PMC6494360 30817251

[B58] Guo C. Manjili M. H. Subjeck J. R. Sarkar D. Fisher P. B. Wang X.-Y. (2013). Therapeutic Cancer Vaccines. Adv. Cancer Res. 119, 421–475. 10.1016/B978-0-12-407190-2.00007-1 23870514 PMC3721379

[B59] Hailemichael Y. Dai Z. Jaffarzad N. Ye Y. Medina M. A. Huang X.-F. (2013). Persistent Antigen at Vaccination Sites Induces Tumor-specific CD8+ T Cell Sequestration, Dysfunction and Deletion. Nat. Med. 19 (4), 465–472. 10.1038/nm.3105 23455713 PMC3618499

[B60] Hammerich L. Binder A. Brody J. D. (2015). In Situvaccination: Cancer Immunotherapy Both Personalizedandoff-The-Shelf. Mol. Oncol. 9 (10), 1966–1981. 10.1016/j.molonc.2015.10.016 26632446 PMC5528727

[B61] Han F. Y. Thurecht K. J. Whittaker A. K. Smith M. T. (2016a). Bioerodable PLGA-Based Microparticles for Producing Sustained-Release Drug Formulations and Strategies for Improving Drug Loading. Front. Pharmacol. 7, 185. 10.3389/fphar.2016.00185 27445821 PMC4923250

[B62] Han L. Xue J. Wang L. Peng K. Zhang Z. Gong T. (2016b). An Injectable, Low-Toxicity Phospholipid-Based Phase Separation Gel that Induces Strong and Persistent Immune Responses in Mice. Biomaterials 105, 185–194. 10.1016/j.biomaterials.2016.08.007 27522253

[B63] Hori Y. Stern P. J. Hynes R. O. Irvine D. J. (2009). Engulfing Tumors with Synthetic Extracellular Matrices for Cancer Immunotherapy. Biomaterials 30 (35), 6757–6767. 10.1016/j.biomaterials.2009.08.037 19766305 PMC2788234

[B64] Hossain M. K. Ahmed T. Bhusal P. Subedi R. K. Salahshoori I. Soltani M. (2020). Microneedle Systems for Vaccine Delivery: the Story So Far. Expert Rev. Vaccin. 19 (12), 1153–1166. 10.1080/14760584.2020.1874928 33427523

[B65] Hou Y. Liu R. Hong X. Zhang Y. Bai S. Luo X. (2021). Engineering a Sustained Release Vaccine with a Pathogen-Mimicking Manner for Robust and Durable Immune Responses. J. Controlled Release 333, 162–175. 10.1016/j.jconrel.2021.03.037 33794269

[B66] Hu Z. Ott P. A. Wu C. J. (2018). Towards Personalized, Tumour-specific, Therapeutic Vaccines for Cancer. Nat. Rev. Immunol. 18 (3), 168–182. 10.1038/nri.2017.131 29226910 PMC6508552

[B67] Huang P. Wang X. Liang X. Yang J. Zhang C. Kong D. (2019). Nano-, Micro-, and Macroscale Drug Delivery Systems for Cancer Immunotherapy. Acta Biomater. 85, 1–26. 10.1016/j.actbio.2018.12.028 30579043

[B68] Huang X. Williams J. Z. Chang R. Li Z. Burnett C. E. Hernandez-Lopez R. (2020). DNA Scaffolds Enable Efficient and Tunable Functionalization of Biomaterials for Immune Cell Modulation. Nat. Nanotechnol. 16 (2), 214–223. 10.1038/s41565-020-00813-z 33318641 PMC7878327

[B69] Hwang M. P. Fecek R. J. Qin T. Storkus W. J. Wang Y. (2020). Single Injection of IL-12 Coacervate as an Effective Therapy against B16-F10 Melanoma in Mice. J. Controlled Release 318, 270–278. 10.1016/j.jconrel.2019.12.035 PMC7045464 31866503

[B70] Iezzi G. Karjalainen K. Lanzavecchia A. (1998). The Duration of Antigenic Stimulation Determines the Fate of Naive and Effector T Cells. Immunity 8 (1), 89–95. 10.1016/s1074-7613(00)80461-6 9462514

[B71] Irvine D. J. Aung A. Silva M. (2020). Controlling Timing and Location in Vaccines. Adv. Drug Deliv. Rev. 158, 91–115. 10.1016/j.addr.2020.06.019 32598970 PMC7318960

[B72] Jackaman C. Nelson D. J. (2012). Intratumoral Interleukin-2/agonist CD40 Antibody Drives CD4+-independent Resolution of Treated-Tumors and CD4+-dependent Systemic and Memory Responses. Cancer Immunol. Immunother. 61 (4), 549–560. 10.1007/s00262-011-1120-5 22002241 PMC11029634

[B73] Jacobs J. J. Snackey C. Geldof A. A. Characiejus D. Van Moorselaar R. J. Den Otter W. (2014). Inefficacy of Therapeutic Cancer Vaccines and Proposed Improvements. Casus of Prostate Cancer. Anticancer Res. 34 (6), 2689–2700. 24922629

[B74] Janku F. Zhang H. H. Pezeshki A. Goel S. Murthy R. Wang-Gillam A. (2021). Intratumoral Injection of *Clostridium* Novyi-NT Spores in Patients with Treatment-Refractory Advanced Solid Tumors. Clin. Cancer Res. 27 (1), 96–106. 10.1158/1078-0432.CCR-20-2065 33046513

[B75] Jiang H. Wang Q. Sun X. (2017). Lymph Node Targeting Strategies to Improve Vaccination Efficacy. J. Controlled Release 267, 47–56. 10.1016/j.jconrel.2017.08.009 28818619

[B76] Jin X. Zhu D. D. Chen B. Z. Ashfaq M. Guo X. D. (2018a). Insulin Delivery Systems Combined with Microneedle Technology. Adv. Drug Deliv. Rev. 127, 119–137. 10.1016/j.addr.2018.03.011 29604374

[B77] Jin X. Zhu D. D. Chen B. Z. Ashfaq M. Guo X. D. (2018b). Insulin Delivery Systems Combined with Microneedle Technology. Adv. Drug Deliv. Rev. 127, 119–137. 10.1016/j.addr.2018.03.011 29604374

[B78] Johansen P. Storni T. Rettig L. Qiu Z. Der-Sarkissian A. Smith K. A. (2008). Antigen Kinetics Determines Immune Reactivity. Pnas 105 (13), 5189–5194. 10.1073/pnas.0706296105 18362362 PMC2278203

[B79] Joshi V. B. Geary S. M. Gross B. P. Wongrakpanich A. Norian L. A. Salem A. K. (2014). Tumor Lysate-Loaded Biodegradable Microparticles as Cancer Vaccines. Expert Rev. Vaccin. 13 (1), 9–15. 10.1586/14760584.2014.851606 PMC3968791 24219096

[B80] Jung H. Jang H.-E. Kang Y. Y. Song J. Mok H. (2019). PLGA Microspheres Coated with Cancer Cell-Derived Vesicles for Improved Internalization into Antigen-Presenting Cells and Immune Stimulation. Bioconjug. Chem. 30 (6), 1690–1701. 10.1021/acs.bioconjchem.9b00240 31018638

[B81] Kanchan V. Katare Y. K. Panda A. K. (2009). Memory Antibody Response from Antigen Loaded Polymer Particles and the Effect of Antigen Release Kinetics. Biomaterials 30 (27), 4763–4776. 10.1016/j.biomaterials.2009.05.075 19540583

[B82] Kanwar N. Sinha V. R. (2019). *In situ* forming Depot as Sustained-Release Drug Delivery Systems. Crit. Rev. Ther. Drug Carrier Syst. 36 (2), 93–136. 10.1615/CritRevTherDrugCarrierSyst.2018025013 30806210

[B83] Keler T. He L. Ramakrishna V. Champion B. (2007). Antibody-targeted Vaccines. Oncogene 26 (25), 3758–3767. 10.1038/sj.onc.1210375 17530028

[B84] Kim H. Seong K.-Y. Lee J. H. Park W. Yang S. Y. Hahn S. K. (2019). Biodegradable Microneedle Patch Delivering Antigenic Peptide-Hyaluronate Conjugate for Cancer Immunotherapy. ACS Biomater. Sci. Eng. 5 (10), 5150–5158. 10.1021/acsbiomaterials.9b00961 33455221

[B85] Kim J. Li W. A. Choi Y. Lewin S. A. Verbeke C. S. Dranoff G. (2015). Injectable, Spontaneously Assembling, Inorganic Scaffolds Modulate Immune Cells in vivo and Increase Vaccine Efficacy. Nat. Biotechnol. 33 (1), 64–72. 10.1038/nbt.3071 25485616 PMC4318563

[B86] Kim N. W. Kim S.-Y. Lee J. E. Yin Y. Lee J. H. Lim S. Y. (2018). Enhanced Cancer Vaccination by In Situ Nanomicelle-Generating Dissolving Microneedles. ACS Nano 12 (10), 9702–9713. 10.1021/acsnano.8b04146 30141896

[B87] Knorr D. A. Dahan R. Ravetch J. V. (2018). Toxicity of an Fc-Engineered Anti-CD40 Antibody Is Abrogated by Intratumoral Injection and Results in Durable Antitumor Immunity. Proc. Natl. Acad. Sci. USA 115 (43), 11048–11053. 10.1073/pnas.1810566115 30297432 PMC6205479

[B88] Koerner J. Horvath D. Groettrup M. (2019). Harnessing Dendritic Cells for Poly (D,L-lactide-co-glycolide) Microspheres (PLGA MS)-mediated Anti-tumor Therapy. Front. Immunol. 10, 707. 10.3389/fimmu.2019.00707 31024545 PMC6460768

[B89] Kordalivand N. Tondini E. Lau C. Y. J. Vermonden T. Mastrobattista E. Hennink W. E. (2019). Cationic Synthetic Long Peptides-Loaded Nanogels: an Efficient Therapeutic Vaccine Formulation for Induction of T-Cell Responses. J. Controlled Release 315, 114–125. 10.1016/j.jconrel.2019.10.048 31672626

[B90] Korupalli C. Pan W.-Y. Yeh C.-Y. Chen P.-M. Mi F.-L. Tsai H.-W. (2019). Single-injecting, Bioinspired Nanocomposite Hydrogel that Can Recruit Host Immune Cells In Situ to Elicit Potent and Long-Lasting Humoral Immune Responses. Biomaterials 216, 119268. 10.1016/j.biomaterials.2019.119268 31226570

[B91] Kuol N. Stojanovska L. Nurgali K. Apostolopoulos V. (2017). The Mechanisms Tumor Cells Utilize to Evade the Host's Immune System. Maturitas 105, 8–15. 10.1016/j.maturitas.2017.04.014 28477990

[B92] Labanieh L. Majzner R. G. Mackall C. L. (2018). Programming CAR-T Cells to Kill Cancer. Nat. Biomed. Eng. 2 (6), 377–391. 10.1038/s41551-018-0235-9 31011197

[B93] Leach D. G. Young S. Hartgerink J. D. (2019). Advances in Immunotherapy Delivery from Implantable and Injectable Biomaterials. Acta Biomater. 88, 15–31. 10.1016/j.actbio.2019.02.016 30771535 PMC6632081

[B94] Lei K. Tang L. (2019). Surgery-free Injectable Macroscale Biomaterials for Local Cancer Immunotherapy. Biomater. Sci. 7 (3), 733–749. 10.1039/c8bm01470a 30637428

[B95] Leone M. Mönkäre J. Bouwstra J. A. Kersten G. (2017). Dissolving Microneedle Patches for Dermal Vaccination. Pharm. Res. 34 (11), 2223–2240. 10.1007/s11095-017-2223-2 28718050 PMC5643353

[B96] Li A. W. Sobral M. C. Badrinath S. Choi Y. Graveline A. Stafford A. G. (2018). A Facile Approach to Enhance Antigen Response for Personalized Cancer Vaccination. Nat. Mater 17 (6), 528–534. 10.1038/s41563-018-0028-2 29507416 PMC5970019

[B97] Li F. Lyu D. Liu S. Guo W. (2020). DNA Hydrogels and Microgels for Biosensing and Biomedical Applications. Adv. Mater. 32 (3), e1806538. 10.1002/adma.201806538 31379017

[B98] Li J. Mooney D. J. (2016). Designing Hydrogels for Controlled Drug Delivery. Nat. Rev. Mater. 1 (12), 16071. 10.1038/natrevmats.2016.71 29657852 PMC5898614

[B99] Li W. A. Lu B. Y. Gu L. Choi Y. Kim J. Mooney D. J. (2016a). The Effect of Surface Modification of Mesoporous Silica Micro-rod Scaffold on Immune Cell Activation and Infiltration. Biomaterials 83, 249–256. 10.1016/j.biomaterials.2016.01.026 26784009 PMC4754159

[B100] Li Y. Yang H. Y. Lee D. S. (2021). Advances in Biodegradable and Injectable Hydrogels for Biomedical Applications. J. Controlled Release 330, 151–160. 10.1016/j.jconrel.2020.12.008 33309972

[B101] Li Z. Xiong F. He J. Dai X. Wang G. (2016b). Surface-functionalized, pH-Responsive Poly(lactic-Co-Glycolic Acid)-Based Microparticles for Intranasal Vaccine Delivery: Effect of Surface Modification with Chitosan and Mannan. Eur. J. Pharmaceutics Biopharmaceutics 109, 24–34. 10.1016/j.ejpb.2016.08.012 27569030

[B102] Lin C.-Y. Lin S.-J. Yang Y.-C. Wang D.-Y. Cheng H.-F. Yeh M.-K. (2015). Biodegradable Polymeric Microsphere-Based Vaccines and Their Applications in Infectious Diseases. Hum. Vaccin. Immunother. 11 (3), 650–656. 10.1080/21645515.2015.1009345 25839217 PMC4514183

[B103] Lin Y.-X. Wang Y. Blake S. Yu M. Mei L. Wang H. (2020). RNA Nanotechnology-Mediated Cancer Immunotherapy. Theranostics 10 (1), 281–299. 10.7150/thno.35568 31903120 PMC6929632

[B104] Liu H. Moynihan K. D. Zheng Y. Szeto G. L. Li A. V. Huang B. (2014). Structure-based Programming of Lymph-Node Targeting in Molecular Vaccines. Nature 507 (7493), 519–522. 10.1038/nature12978 24531764 PMC4069155

[B105] Liu H. Shi X. Wu D. Kahsay Khshen F. Deng L. Dong A. (2019a). Injectable, Biodegradable, Thermosensitive Nanoparticles-Aggregated Hydrogel with Tumor-specific Targeting, Penetration, and Release for Efficient Postsurgical Prevention of Tumor Recurrence. ACS Appl. Mater. Inter. 11 (22), 19700–19711. 10.1021/acsami.9b01987 31070356

[B106] Liu W.-L. Zou M.-Z. Liu T. Zeng J.-Y. Li X. Yu W.-Y. (2019b). Cytomembrane Nanovaccines Show Therapeutic Effects by Mimicking Tumor Cells and Antigen Presenting Cells. Nat. Commun. 10 (1), 3199. 10.1038/s41467-019-11157-1 31324770 PMC6642123

[B107] Lopes A. Vandermeulen G. Préat V. (2019). Cancer DNA Vaccines: Current Preclinical and Clinical Developments and Future Perspectives. J. Exp. Clin. Cancer Res. 38 (1), 146. 10.1186/s13046-019-1154-7 30953535 PMC6449928

[B108] Lu Y. Aimetti A. A. Langer R. Gu Z. (2017). Bioresponsive Materials. Nat. Rev. Mater. 2, 16075. 10.1038/natrevmats.2016.75

[B109] Lybaert L. Vermaelen K. De Geest B. G. Nuhn L. (2018). Immunoengineering through Cancer Vaccines - A Personalized and Multi-step Vaccine Approach towards Precise Cancer Immunity. J. Controlled Release 289, 125–145. 10.1016/j.jconrel.2018.09.009 30223044

[B110] Mata E. Igartua M. Patarroyo M. E. Pedraz J. L. Hernández R. M. (2011). Enhancing Immunogenicity to PLGA Microparticulate Systems by Incorporation of Alginate and RGD-Modified Alginate. Eur. J. Pharm. Sci. 44 (1-2), 32–40. 10.1016/j.ejps.2011.05.015 21699977

[B111] Matson V. Fessler J. Bao R. Chongsuwat T. Zha Y. Alegre M.-L. (2018). The Commensal Microbiome Is Associated with Anti-PD-1 Efficacy in Metastatic Melanoma Patients. Science 359 (6371), 104–108. 10.1126/science.aao3290 29302014 PMC6707353

[B112] McHugh K. J. Guarecuco R. Langer R. Jaklenec A. (2015). Single-injection Vaccines: Progress, Challenges, and Opportunities. J. Controlled Release 219, 596–609. 10.1016/j.jconrel.2015.07.029 26254198

[B113] McNamara M. A. Nair S. K. Holl E. K. (2015). RNA-based Vaccines in Cancer Immunotherapy. J. Immunol. Res. 2015, 794528. 10.1155/2015/794528 26665011 PMC4668311

[B114] Mestas J. Hughes C. C. W. (2004). Of Mice and Not Men: Differences between Mouse and Human Immunology. J. Immunol. 172 (5), 2731–2738. 10.4049/jimmunol.172.5.2731 14978070

[B115] Mills B. N. Connolly K. A. Ye J. Murphy J. D. Uccello T. P. Han B. J. (2019). Stereotactic Body Radiation and Interleukin-12 Combination Therapy Eradicates Pancreatic Tumors by Repolarizing the Immune Microenvironment. Cel Rep. 29 (2), 406–421. e405. 10.1016/j.celrep.2019.08.095 PMC6919969 31597100

[B116] Min Y. Roche K. C. Tian S. Eblan M. J. McKinnon K. P. Caster J. M. (2017). Antigen-capturing Nanoparticles Improve the Abscopal Effect and Cancer Immunotherapy. Nat. Nanotech 12 (9), 877–882. 10.1038/nnano.2017.113 PMC5587366 28650437

[B117] Mueller M. Reichardt W. Koerner J. Groettrup M. (2012). Coencapsulation of Tumor Lysate and CpG-ODN in PLGA-Microspheres Enables Successful Immunotherapy of Prostate Carcinoma in TRAMP Mice. J. Controlled Release 162 (1), 159–166. 10.1016/j.jconrel.2012.06.015 22709589

[B118] Nejman D. Livyatan I. Fuks G. Gavert N. Zwang Y. Geller L. T. (2020). The Human Tumor Microbiome Is Composed of Tumor Type-specific Intracellular Bacteria. Science 368 (6494), 973–980. 10.1126/science.aay9189 32467386 PMC7757858

[B119] Nguyen T. L. Yin Y. Choi Y. Jeong J. H. Kim J. (2020). Enhanced Cancer DNA Vaccine via Direct Transfection to Host Dendritic Cells Recruited in Injectable Scaffolds. ACS Nano 14 (9), 11623–11636. 10.1021/acsnano.0c04188 32808762

[B120] Niu L. Chu L. Y. Burton S. A. Hansen K. J. Panyam J. (2019). Intradermal Delivery of Vaccine Nanoparticles Using Hollow Microneedle Array Generates Enhanced and Balanced Immune Response. J. Controlled Release 294, 268–278. 10.1016/j.jconrel.2018.12.026 30572036

[B121] Noguchi M. Arai G. Egawa S. Ohyama C. Naito S. Matsumoto K. (2020). Mixed 20-peptide Cancer Vaccine in Combination with Docetaxel and Dexamethasone for Castration-Resistant Prostate Cancer: a Randomized Phase II Trial. Cancer Immunol. Immunother. 69 (5), 847–857. 10.1007/s00262-020-02498-8 32025848 PMC7183507

[B122] Obeid J. Hu Y. Slingluff C. L. Jr. (2015). Vaccines, Adjuvants, and Dendritic Cell Activators-Current Status and Future Challenges. Semin. Oncol. 42 (4), 549–561. 10.1053/j.seminoncol.2015.05.006 26320060 PMC4621212

[B123] Oberli M. A. Reichmuth A. M. Dorkin J. R. Mitchell M. J. Fenton O. S. Jaklenec A. (2017). Lipid Nanoparticle Assisted mRNA Delivery for Potent Cancer Immunotherapy. Nano Lett. 17 (3), 1326–1335. 10.1021/acs.nanolett.6b03329 28273716 PMC5523404

[B124] Oh E. Oh J.-E. Hong J. Chung Y. Lee Y. Park K. D. (2017). Optimized Biodegradable Polymeric Reservoir-Mediated Local and Sustained Co-delivery of Dendritic Cells and Oncolytic Adenovirus Co-expressing IL-12 and GM-CSF for Cancer Immunotherapy. J. Controlled Release 259, 115–127. 10.1016/j.jconrel.2017.03.028 28336378

[B125] Ouyang X. Telli M. L. Wu J. C. (2019). Induced Pluripotent Stem Cell-Based Cancer Vaccines. Front. Immunol. 10, 1510. 10.3389/fimmu.2019.01510 31338094 PMC6628907

[B126] Ozao-Choy J. Lee D. J. Faries M. B. (2014). Melanoma Vaccines. Surg. Clin. North America 94 (5), 1017–1030. 10.1016/j.suc.2014.07.005 PMC4173123 25245965

[B127] Pardi N. Hogan M. J. Porter F. W. Weissman D. (2018). mRNA Vaccines - a New Era in Vaccinology. Nat. Rev. Drug Discov. 17 (4), 261–279. 10.1038/nrd.2017.243 29326426 PMC5906799

[B128] Park K. Skidmore S. Hadar J. Garner J. Park H. Otte A. (2019). Injectable, Long-Acting PLGA Formulations: Analyzing PLGA and Understanding Microparticle Formation. J. Controlled Release 304, 125–134. 10.1016/j.jconrel.2019.05.003 31071374

[B129] Patel R. B. Ye M. Carlson P. M. Jaquish A. Zangl L. Ma B. (2019). Development of an *In Situ* Cancer Vaccine via Combinational Radiation and Bacterial‐Membrane‐Coated Nanoparticles. Adv. Mater. 31 (43), e1902626. 10.1002/adma.201902626 31523868 PMC6810793

[B130] Perez C. R. De Palma M. (2019). Engineering Dendritic Cell Vaccines to Improve Cancer Immunotherapy. Nat. Commun. 10 (1), 5408. 10.1038/s41467-019-13368-y 31776331 PMC6881351

[B131] Peris I. Langer R. S. (1979). A Single-step Immunization by Sustained Antigen Release. J. Immunol. Methods 28 (1), 193–197. 10.1016/0022-1759(79)90341-7 469267

[B132] Phuengkham H. Song C. Um S. H. Lim Y. T. (2018). Implantable Synthetic Immune Niche for Spatiotemporal Modulation of Tumor-Derived Immunosuppression and Systemic Antitumor Immunity: Postoperative Immunotherapy. Adv. Mater. 30 (18), e1706719. 10.1002/adma.201706719 29572968

[B133] Pilla L. Ferrone S. Maccalli C. (2018). Methods for Improving the Immunogenicity and Efficacy of Cancer Vaccines. Expert Opin. Biol. Ther. 18 (7), 765–784. 10.1080/14712598.2018.1485649 29874943 PMC8670419

[B134] Pol J. Vacchelli E. Aranda F. Castoldi F. Eggermont A. Cremer I. (2015). Trial Watch: Immunogenic Cell Death Inducers for Anticancer Chemotherapy. Oncoimmunol. 4 (4), e1008866. 10.1080/2162402X.2015.1008866 PMC4485780 26137404

[B135] Pradhan P. Qin H. Leleux J. A. Gwak D. Sakamaki I. Kwak L. W. (2014). The Effect of Combined IL10 siRNA and CpG ODN as Pathogen-Mimicking Microparticles on Th1/Th2 Cytokine Balance in Dendritic Cells and Protective Immunity against B Cell Lymphoma. Biomaterials 35 (21), 5491–5504. 10.1016/j.biomaterials.2014.03.039 24720881 PMC4747034

[B136] Qu J. Zhao X. Ma P. X. Guo B. (2018). Injectable Antibacterial Conductive Hydrogels with Dual Response to an Electric Field and pH for Localized "smart" Drug Release. Acta Biomater. 72, 55–69. 10.1016/j.actbio.2018.03.018 29555459

[B137] Rahimian S. Fransen M. F. Kleinovink J. W. Amidi M. Ossendorp F. Hennink W. E. (2015). Polymeric Microparticles for Sustained and Local Delivery of antiCD40 and antiCTLA-4 in Immunotherapy of Cancer. Biomaterials 61, 33–40. 10.1016/j.biomaterials.2015.04.043 25993015

[B138] Rehman H. Silk A. W. Kane M. P. Kaufman H. L. (2016). Into the Clinic: Talimogene Laherparepvec (T-VEC), a First-In-Class Intratumoral Oncolytic Viral Therapy. J. Immunotherapy Cancer 4, 53. 10.1186/s40425-016-0158-5 PMC5029010 27660707

[B139] Ribas A. Weber J. S. Chmielowski B. Comin-Anduix B. Lu D. Douek M. (2011). Intra-lymph Node Prime-Boost Vaccination against Melan A and Tyrosinase for the Treatment of Metastatic Melanoma: Results of a Phase 1 Clinical Trial. Clin. Cancer Res. 17 (9), 2987–2996. 10.1158/1078-0432.Ccr-10-3272 21385924

[B140] Riley R. S. June C. H. Langer R. Mitchell M. J. (2019). Delivery Technologies for Cancer Immunotherapy. Nat. Rev. Drug Discov. 18 (3), 175–196. 10.1038/s41573-018-0006-z 30622344 PMC6410566

[B141] Rosalia R. A. Quakkelaar E. D. Redeker A. Khan S. Camps M. Drijfhout J. W. (2013). Dendritic Cells Process Synthetic Long Peptides Better Than Whole Protein, Improving Antigen Presentation and T-Cell Activation. Eur. J. Immunol. 43 (10), 2554–2565. 10.1002/eji.201343324 23836147

[B142] Russell S. J. Barber G. N. (2018). Oncolytic Viruses as Antigen-Agnostic Cancer Vaccines. Cancer Cell 33 (4), 599–605. 10.1016/j.ccell.2018.03.011 29634947 PMC5918693

[B143] Sabado R. L. Balan S. Bhardwaj N. (2017). Dendritic Cell-Based Immunotherapy. Cell Res 27 (1), 74–95. 10.1038/cr.2016.157 28025976 PMC5223236

[B144] Schineis P. Kotkowska Z. K. Vogel-Kindgen S. Friess M. C. Theisen M. Schwyter D. (2021). Photochemical Internalization (PCI)-mediated Activation of CD8 T Cells Involves Antigen Uptake and CCR7-Mediated Transport by Migratory Dendritic Cells to Draining Lymph Nodes. J. Controlled Release 332, 96–108. 10.1016/j.jconrel.2021.02.014 33609623

[B145] Schlom J. Hodge J. W. Palena C. Tsang K.-Y. Jochems C. Greiner J. W. (2014). Therapeutic Cancer Vaccines. Adv. Cancer Res. 121, 67–124. 10.1016/B978-0-12-800249-0.00002-0 24889529 PMC6324585

[B146] Shae D. Baljon J. J. Wehbe M. Becker K. W. Sheehy T. L. Wilson J. T. (2019). At the Bench: Engineering the Next Generation of Cancer Vaccines. J. Leukoc. Biol. 108 (4), 1435–1453. 10.1002/jlb.5bt0119-016r 31430398

[B147] Shafiee A. Atala A. (2017). Tissue Engineering: toward a New Era of Medicine. Annu. Rev. Med. 68, 29–40. 10.1146/annurev-med-102715-092331 27732788

[B148] Sharma P. Allison J. P. (2015). Immune Checkpoint Targeting in Cancer Therapy: toward Combination Strategies with Curative Potential. Cell 161 (2), 205–214. 10.1016/j.cell.2015.03.030 25860605 PMC5905674

[B149] Sheen M. R. Fiering S. (2019). *In situ* vaccination: Harvesting Low Hanging Fruit on the Cancer Immunotherapy Tree. WIREs Nanomed Nanobiotechnol 11 (1), e1524. 10.1002/wnan.1524 29667346

[B150] Silva A. L. Soema P. C. Slütter B. Ossendorp F. Jiskoot W. (2016). PLGA Particulate Delivery Systems for Subunit Vaccines: Linking Particle Properties to Immunogenicity. Hum. Vaccin. Immunother. 12 (4), 1056–1069. 10.1080/21645515.2015.1117714 26752261 PMC4962933

[B151] Sivakumar S. M. Safhi M. M. Kannadasan M. Sukumaran N. (2011). Vaccine Adjuvants - Current Status and Prospects on Controlled Release Adjuvancity. Saudi Pharm. J. 19 (4), 197–206. 10.1016/j.jsps.2011.06.003 23960760 PMC3744968

[B152] Song C. Phuengkham H. Kim Y. S. Dinh V. V. Lee I. Shin I. W. (2019). Syringeable Immunotherapeutic Nanogel Reshapes Tumor Microenvironment and Prevents Tumor Metastasis and Recurrence. Nat. Commun. 10 (1), 3745. 10.1038/s41467-019-11730-8 31431623 PMC6702226

[B153] Song H. Huang P. Niu J. Shi G. Zhang C. Kong D. (2018). Injectable Polypeptide Hydrogel for Dual-Delivery of Antigen and TLR3 Agonist to Modulate Dendritic Cells in vivo and Enhance Potent Cytotoxic T-Lymphocyte Response against Melanoma. Biomaterials 159, 119–129. 10.1016/j.biomaterials.2018.01.004 29324304

[B154] Sprooten J. Ceusters J. Coosemans A. Agostinis P. De Vleeschouwer S. Zitvogel L. (2019). Trial Watch: Dendritic Cell Vaccination for Cancer Immunotherapy. Oncoimmunol. 8 (11), e1638212. 10.1080/2162402x.2019.1638212 PMC6791419 31646087

[B155] Stanley M. (2017). Tumour Virus Vaccines: Hepatitis B Virus and Human Papillomavirus. Phil. Trans. R. Soc. B 372 (1732), 20160268. 10.1098/rstb.2016.0268 28893935 PMC5597735

[B156] Stephan S. B. Taber A. M. Jileaeva I. Pegues E. P. Sentman C. L. Stephan M. T. (2015). Biopolymer Implants Enhance the Efficacy of Adoptive T-Cell Therapy. Nat. Biotechnol. 33 (1), 97–101. 10.1038/nbt.3104 25503382 PMC4289408

[B157] Subbiah V. Murthy R. Hong D. S. Prins R. M. Hosing C. Hendricks K. (2018). Cytokines Produced by Dendritic Cells Administered Intratumorally Correlate with Clinical Outcome in Patients with Diverse Cancers. Clin. Cancer Res. 24 (16), 3845–3856. 10.1158/1078-0432.CCR-17-2707 30018119 PMC6449174

[B158] Tanyi J. L. Bobisse S. Ophir E. Tuyaerts S. Roberti A. Genolet R. (2018). Personalized Cancer Vaccine Effectively Mobilizes Antitumor T Cell Immunity in Ovarian Cancer. Sci. Transl. Med. 10 (436), eaao5931. 10.1126/scitranslmed.aao5931 29643231

[B159] Théry C. Ostrowski M. Segura E. (2009). Membrane Vesicles as Conveyors of Immune Responses. Nat. Rev. Immunol. 9 (8), 581–593. 10.1038/nri2567 19498381

[B160] Twumasi-Boateng K. Pettigrew J. L. Kwok Y. Y. E. Bell J. C. Nelson B. H. (2018). Oncolytic Viruses as Engineering Platforms for Combination Immunotherapy. Nat. Rev. Cancer 18 (7), 419–432. 10.1038/s41568-018-0009-4 29695749

[B161] Umeki Y. Mohri K. Kawasaki Y. Watanabe H. Takahashi R. Takahashi Y. (2015). Induction of Potent Antitumor Immunity by Sustained Release of Cationic Antigen from a DNA-Based Hydrogel with Adjuvant Activity. Adv. Funct. Mater. 25 (36), 5758–5767. 10.1002/adfm.201502139

[B162] Uppu D. S. S. M. Turvey M. E. Sharif A. R. M. Bidet K. He Y. Ho V. (2020). Temporal Release of a Three-Component Protein Subunit Vaccine from Polymer Multilayers. J. Controlled Release 317, 130–141. 10.1016/j.jconrel.2019.11.022 31756392

[B163] US National Library of Medicine (2021). Dendritic Cell Activating Scaffold in Melanoma. [Online]. Available: https://clinicaltrials.gov/ct2/show/NCT01753089 (Accessed March 10, 2021).

[B164] van der Burg S. H. Arens R. Ossendorp F. van Hall T. Melief C. J. M. (2016). Vaccines for Established Cancer: Overcoming the Challenges Posed by Immune Evasion. Nat. Rev. Cancer 16 (4), 219–233. 10.1038/nrc.2016.16 26965076

[B165] van der Burg S. H. (2018). Correlates of Immune and Clinical Activity of Novel Cancer Vaccines. Semin. Immunol. 39, 119–136. 10.1016/j.smim.2018.04.001 29709421

[B166] van der Maaden K. Heuts J. Camps M. Pontier M. Terwisscha van Scheltinga A. Jiskoot W. (2018). Hollow Microneedle-Mediated Micro-injections of a Liposomal HPV E743-63 Synthetic Long Peptide Vaccine for Efficient Induction of Cytotoxic and T-Helper Responses. J. Controlled Release 269, 347–354. 10.1016/j.jconrel.2017.11.035 29174441

[B167] Vartak A. Sucheck S. (2016). Recent Advances in Subunit Vaccine Carriers. Vaccines 4 (2), 12. 10.3390/vaccines4020012 PMC4931629 27104575

[B168] Wang C. Ye Y. Hochu G. M. Sadeghifar H. Gu Z. (2016). Enhanced Cancer Immunotherapy by Microneedle Patch-Assisted Delivery of Anti-PD1 Antibody. Nano Lett. 16 (4), 2334–2340. 10.1021/acs.nanolett.5b05030 26999507

[B169] Wang H. Mooney D. J. (2018). Biomaterial-assisted Targeted Modulation of Immune Cells in Cancer Treatment. Nat. Mater 17 (9), 761–772. 10.1038/s41563-018-0147-9 30104668

[B170] Wang H. Najibi A. J. Sobral M. C. Seo B. R. Lee J. Y. Wu D. (2020a). Biomaterial-based Scaffold for In Situ Chemo-Immunotherapy to Treat Poorly Immunogenic Tumors. Nat. Commun. 11 (1), 5696. 10.1038/s41467-020-19540-z 33173046 PMC7655953

[B171] Wang T. Wang D. Yu H. Feng B. Zhou F. Zhang H. (2018). A Cancer Vaccine-Mediated Postoperative Immunotherapy for Recurrent and Metastatic Tumors. Nat. Commun. 9 (1), 1532. 10.1038/s41467-018-03915-4 29670088 PMC5906566

[B172] Wang Z. Shang Y. Tan Z. Li X. Li G. Ren C. (2020b). A Supramolecular Protein Chaperone for Vaccine Delivery. Theranostics 10 (2), 657–670. 10.7150/thno.39132 31903143 PMC6929975

[B173] Wei X. Liu L. Li X. Wang Y. Guo X. Zhao J. (2019). Selectively Targeting Tumor-Associated Macrophages and Tumor Cells with Polymeric Micelles for Enhanced Cancer Chemo-Immunotherapy. J. Controlled Release 313, 42–53. 10.1016/j.jconrel.2019.09.021 31629039

[B174] Weiden J. Tel J. Figdor C. G. (2018). Synthetic Immune Niches for Cancer Immunotherapy. Nat. Rev. Immunol. 18 (3), 212–219. 10.1038/nri.2017.89 28853444

[B175] Wu H. Liu S. Xiao L. Dong X. Lu Q. Kaplan D. L. (2016). Injectable and pH-Responsive Silk Nanofiber Hydrogels for Sustained Anticancer Drug Delivery. ACS Appl. Mater. Inter. 8 (27), 17118–17126. 10.1021/acsami.6b04424 27315327

[B176] Yakkala C. Chiang C. L.-L. Kandalaft L. Denys A. Duran R. (2019). Cryoablation and Immunotherapy: an Enthralling Synergy to Confront the Tumors. Front. Immunol. 10, 2283. 10.3389/fimmu.2019.02283 31608067 PMC6769045

[B177] Yan C. Altunbas A. Yucel T. Nagarkar R. P. Schneider J. P. Pochan D. J. (2010). Injectable Solid Hydrogel: Mechanism of Shear-Thinning and Immediate Recovery of Injectable β-hairpin Peptide Hydrogels. Soft Matter 6 (20), 5143–5156. 10.1039/c0sm00642d 21566690 PMC3091287

[B178] Yang P. Song H. Qin Y. Huang P. Zhang C. Kong D. (2018). Engineering Dendritic-Cell-Based Vaccines and PD-1 Blockade in Self-Assembled Peptide Nanofibrous Hydrogel to Amplify Antitumor T-Cell Immunity. Nano Lett. 18 (7), 4377–4385. 10.1021/acs.nanolett.8b01406 29932335

[B179] Yang T. Huang D. Li C. Zhao D. Li J. Zhang M. (2021). Rolling Microneedle Electrode Array (RoMEA) Empowered Nucleic Acid Delivery and Cancer Immunotherapy. Nano Today 36, 101017. 10.1016/j.nantod.2020.101017

[B180] Ye X. Liang X. Chen Q. Miao Q. Chen X. Zhang X. (2019). Surgical Tumor-Derived Personalized Photothermal Vaccine Formulation for Cancer Immunotherapy. ACS Nano 13 (3), 2956–2968. 10.1021/acsnano.8b07371 30789699

[B181] Ye Y. Wang C. Zhang X. Hu Q. Zhang Y. Liu Q. (2017). A Melanin-Mediated Cancer Immunotherapy Patch. Sci. Immunol. 2 (17), eaan5692. 10.1126/sciimmunol.aan5692 29127106

[B182] Yu L. Ding J. (2008). Injectable Hydrogels as Unique Biomedical Materials. Chem. Soc. Rev. 37 (8), 1473–1481. 10.1039/b713009k 18648673

[B183] Yu S. Wang C. Yu J. Wang J. Lu Y. Zhang Y. (2018). Injectable Bioresponsive Gel Depot for Enhanced Immune Checkpoint Blockade. Adv. Mater. 30 (28), e1801527. 10.1002/adma.201801527 29786888

[B184] Zaric M. Lyubomska O. Touzelet O. Poux C. Al-Zahrani S. Fay F. (2013). Skin Dendritic Cell Targeting via Microneedle Arrays Laden with Antigen-Encapsulated Poly-D,l-Lactide-Co-Glycolide Nanoparticles Induces Efficient Antitumor and Antiviral Immune Responses. ACS Nano 7 (3), 2042–2055. 10.1021/nn304235j 23373658 PMC3936823

[B185] Zeng Q. Gammon J. M. Tostanoski L. H. Chiu Y.-C. Jewell C. M. (2017). *In vivo* expansion of Melanoma-specific T Cells Using Microneedle Arrays Coated with Immune-Polyelectrolyte Multilayers. ACS Biomater. Sci. Eng. 3 (2), 195–205. 10.1021/acsbiomaterials.6b00414 28286864 PMC5338335

[B186] Zhang L. Wang W. Wang S. (2015). Effect of Vaccine Administration Modality on Immunogenicity and Efficacy. Expert Rev. Vaccin. 14 (11), 1509–1523. 10.1586/14760584.2015.1081067 PMC4915566 26313239

[B187] Zhang W. Jin X. Li H. Wei C.-x. Wu C.-w. (2019). Onion-structure Bionic Hydrogel Capsules Based on Chitosan for Regulating Doxorubicin Release. Carbohydr. Polym. 209, 152–160. 10.1016/j.carbpol.2019.01.028 30732794

[B188] Zhang W. Wang L. Liu Y. Chen X. Liu Q. Jia J. (2014). Immune Responses to Vaccines Involving a Combined Antigen-Nanoparticle Mixture and Nanoparticle-Encapsulated Antigen Formulation. Biomaterials 35 (23), 6086–6097. 10.1016/j.biomaterials.2014.04.022 24780166

[B189] Zhao H. Zhao B. Li L. Ding K. Xiao H. Zheng C. (2020). Biomimetic Decoy Inhibits Tumor Growth and Lung Metastasis by Reversing the Drawbacks of Sonodynamic Therapy. Adv. Healthc. Mater. 9 (1), e1901335. 10.1002/adhm.201901335 31762228

[B190] Zhao J. Chen Y. Ding Z.-Y. Liu J.-Y. (2019). Safety and Efficacy of Therapeutic Cancer Vaccines Alone or in Combination with Immune Checkpoint Inhibitors in Cancer Treatment. Front. Pharmacol. 10, 1184. 10.3389/fphar.2019.01184 31680963 PMC6798079

[B191] Zhu G. Lynn G. M. Jacobson O. Chen K. Liu Y. Zhang H. (2017). Albumin/vaccine Nanocomplexes that Assemble in vivo for Combination Cancer Immunotherapy. Nat. Commun. 8 (1), 1954. 10.1038/s41467-017-02191-y 29203865 PMC5715147

[B192] Zhu M. Ding X. Zhao R. Liu X. Shen H. Cai C. (2018). Co-delivery of Tumor Antigen and Dual Toll-like Receptor Ligands into Dendritic Cell by Silicon Microparticle Enables Efficient Immunotherapy against Melanoma. J. Controlled Release 272, 72–82. 10.1016/j.jconrel.2018.01.004 PMC5825289 29325699

[B193] Zhu Y. Xue J. Chen W. Bai S. Zheng T. He C. (2020). Albumin-biomineralized Nanoparticles to Synergize Phototherapy and Immunotherapy against Melanoma. J. Controlled Release 322, 300–311. 10.1016/j.jconrel.2020.03.045 32240675

[B194] Zinkernagel R. M. Ehl S. Aichele P. Oehen S. Kündig T. Hengartner H. (1997). Antigen Localisation Regulates Immune Responses in a Dose- and Time-dependent Fashion: a Geographical View of Immune Reactivity. Immunol. Rev. 156, 199–209. 10.1111/j.1600-065x.1997.tb00969.x 9176709

